# Supplementary material for: Benchmark of cellular deconvolution methods using a multi-assay dataset from postmortem human prefrontal cortex
Source: Genome Biol. 2025 Apr 7;26:88. doi: 10.1186/s13059-025-03552-3 (PMC11978107; doi:10.1186/s13059-025-03552-3)
Supplement: Supplementary file 1 — Additional file 1: Contains Fig. S1 to S33 along with their captions. These are the titles: Fig. S1. Schematic of assays performed on each tissue block. Fig. S2. Bulk RNA-seq data Quality Control. Fig. S3. Bulk RNA-seq data Quality Control Principal Component Analysis. Fig. S4. Bulk RNA-seq data Principal Component Analysis. Fig. S5. Volcano plots for RNA extraction Differential Gene Expression analysis. Fig. S6. UpSet plots for Differentially Quantified Genes. Fig. S7. Biotypes of expressed genes in bulk and snRNA-seq datasets. Fig. S8. Enrichment of gene ontology cellular component terms in DQGs. Fig. S9. Representative fluorescence images and corresponding hex plots of all RNAScope/IF circle combination samples. Fig. S10. Representative fluorescence images and corresponding hex plots of all RNAScope/IF star combination samples. Fig. S11. Boxplots of cell type proportions calculated from snRNA-seq and RNAScope/IF data. Fig. S12. Heatmap of the Mean Ratio top25 marker genes for deconvolution. Fig. S13. Volcano plots for library type Differential Gene Expression analysis filtered to Mean Ratio top25 marker genes. Fig. S14. Volcano plots for RNA extraction Differential Gene Expression analysis filtered to Mean Ratio top25 marker genes. Fig. S15. Over-quantification of cell type marker genes in library type and RNA fraction RNA-seq libraries. Fig. S16. Barplots of estimated cell type proportions from deconvolution methods. Fig. S17. Cell composition comparison for Mean Ratio top25 results. Fig. S18. Cell composition results against RNAScope/IF across bulk RNA library type and RNA extractions. Fig. S19. Deconvolution method performance evaluated with Spearman Correlation. Fig. S20. Deconvolution method performance evaluated without Astro. Fig. S21. Variation in estimated neuron proportions across bulk RNA-seq samples from each tissue block. Fig. S22. Oligodendrocyte estimated proportion consistency across polyA and RiboZeroGold. Fig. S23. Deconvolution method performan [file 13059_2025_3552_MOESM1_ESM.pdf]

Supplemental Figures

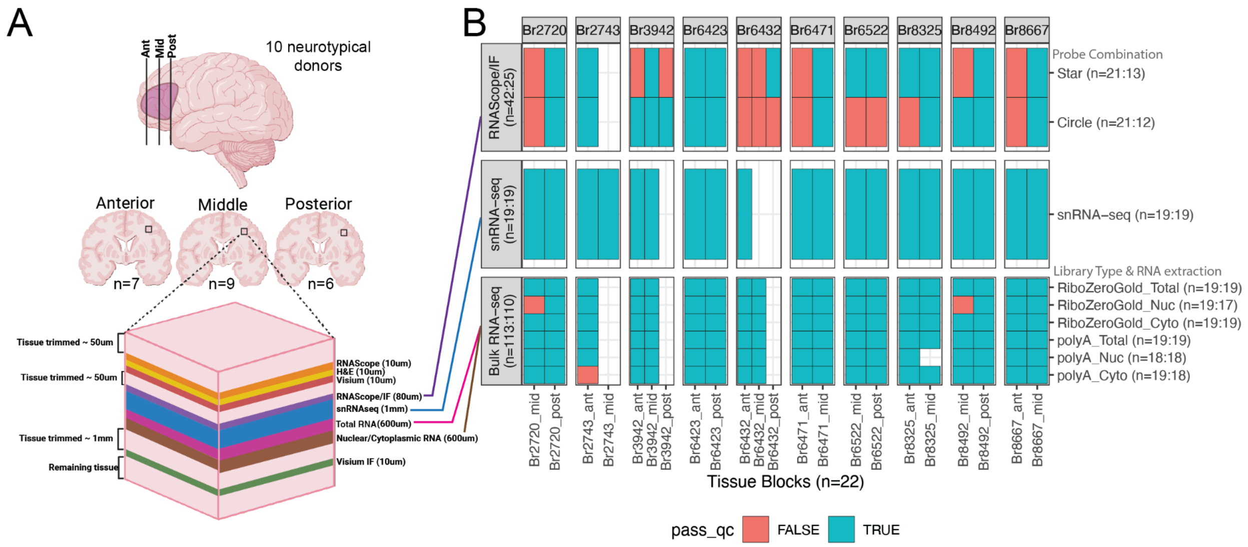

Fig. S1: Schematic of assays performed on each tissue block. A. Schematic of DLPFC dissections and DLPFC tissue block position depicting order of assays completed (n=10 donors; n=22 tissue blocks, including 7 Anterior, 9 Middle, 6 Posterior). Approximately 50 μm of tissue was trimmed to achieve a flat surface for cryosectioning. Next, several ~10 μm sections were collected for anatomical validation (RNAScope/IF, H&E) and Visium experiments [40]. Blocks were stored at -80°C until completion of these assays. At the next cryostat session, blocks were trimmed and ~1 mm of tissue was collected for snRNA-seq (n=19) [40], ~600 μm of tissue was collected for Total RNA extraction for bulk-RNAseq, and ~600 μm of tissue was collected for fractionated RNA extraction for nuclear (Nuc) and cytoplasmic (Cyto) RNA-seq. Finally, four tissue blocks were placed back on the cryostat and trimmed again to obtain a flat surface prior to collecting a ~10 μm Visium-spatial proteogenomics (SPG) tissue section [40]. **B**. Tile plot illustrating which assays and configurations (probe/antibody combination for RNAScope/IF and library type/RNA extraction for RNA-seq) were performed on each tissue block and the sample size for that assay before and after quality control (qc) in the format “(n=before:after)”. The tile is blank if an assay configuration was not performed on the tissue block. The tile is blue if the sample passed qc checks and was included in the analysis. Red tiles are not included in the study.

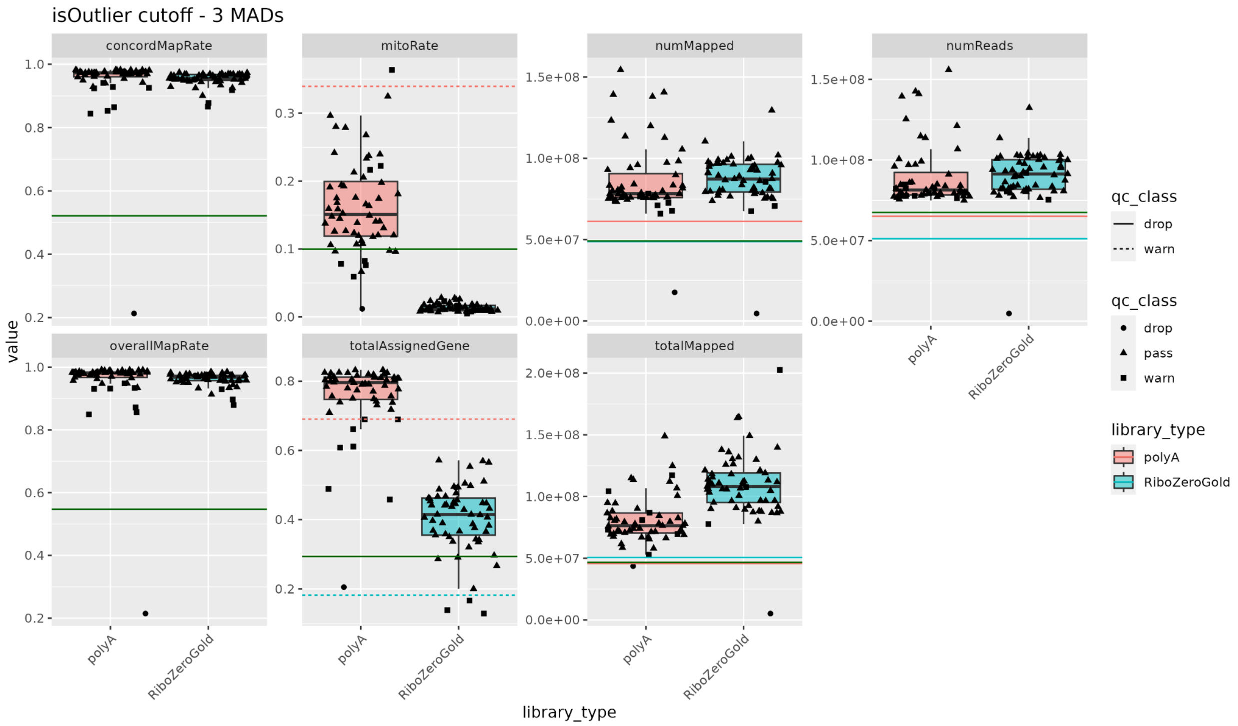

Fig. S2: Bulk RNA-seq data Quality Control. Samples are evaluated for low `concordMapRate`, `numMapped`, `numReads`, `overallMapRate`, `totalAssignedGene`, and `totalMapped` or high `mitoRate`. See the *SPEAQeasy* [62] documentation at https://research.libd.org/SPEAQeasy/outputs.html#quality-metrics for the definition of these variables. Cutoffs (horizontal lines) were determined by a 3 median absolute deviations from the mean (3 MADs) from the distributions for the polyA or RiboZeroGold samples (line color) using isOutlier() from *scran* [41], as well as historic cutoffs from previous LIBD bulk RNA-seq projects (green lines). Based on the distribution of the values, and logic with the QC metrics some were "warning" cutoffs vs. "drop" cutoffs (line type). RNA-seq samples were classified as "drop", "warn", or "pass" based on their relationship to the cutoffs.

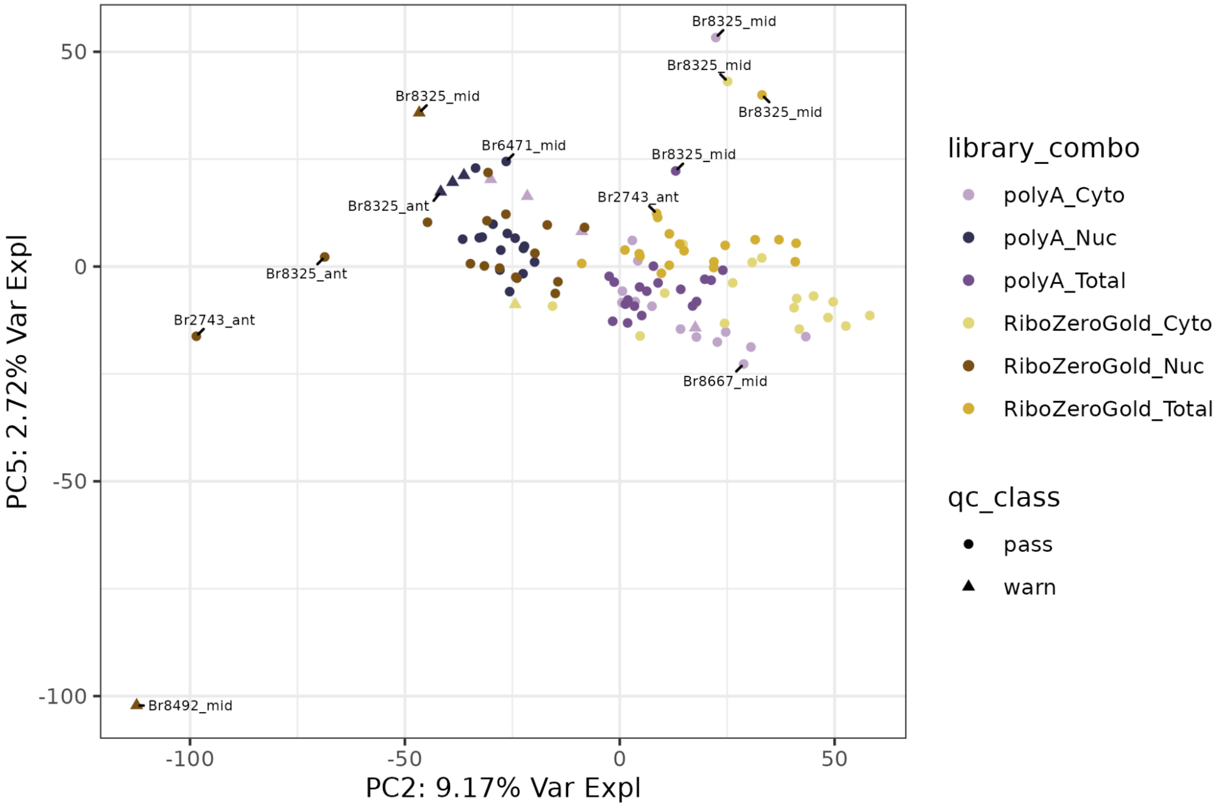

Fig. S3: Bulk RNA-seq data Quality Control Principal Component Analysis (PCA). PC2 versus 5 colored by “library combo” (library type + RNA extraction). The sample `AN00000906_Br8492_Mid_Nuc` was identified as an outlier and removed from downstream analysis.

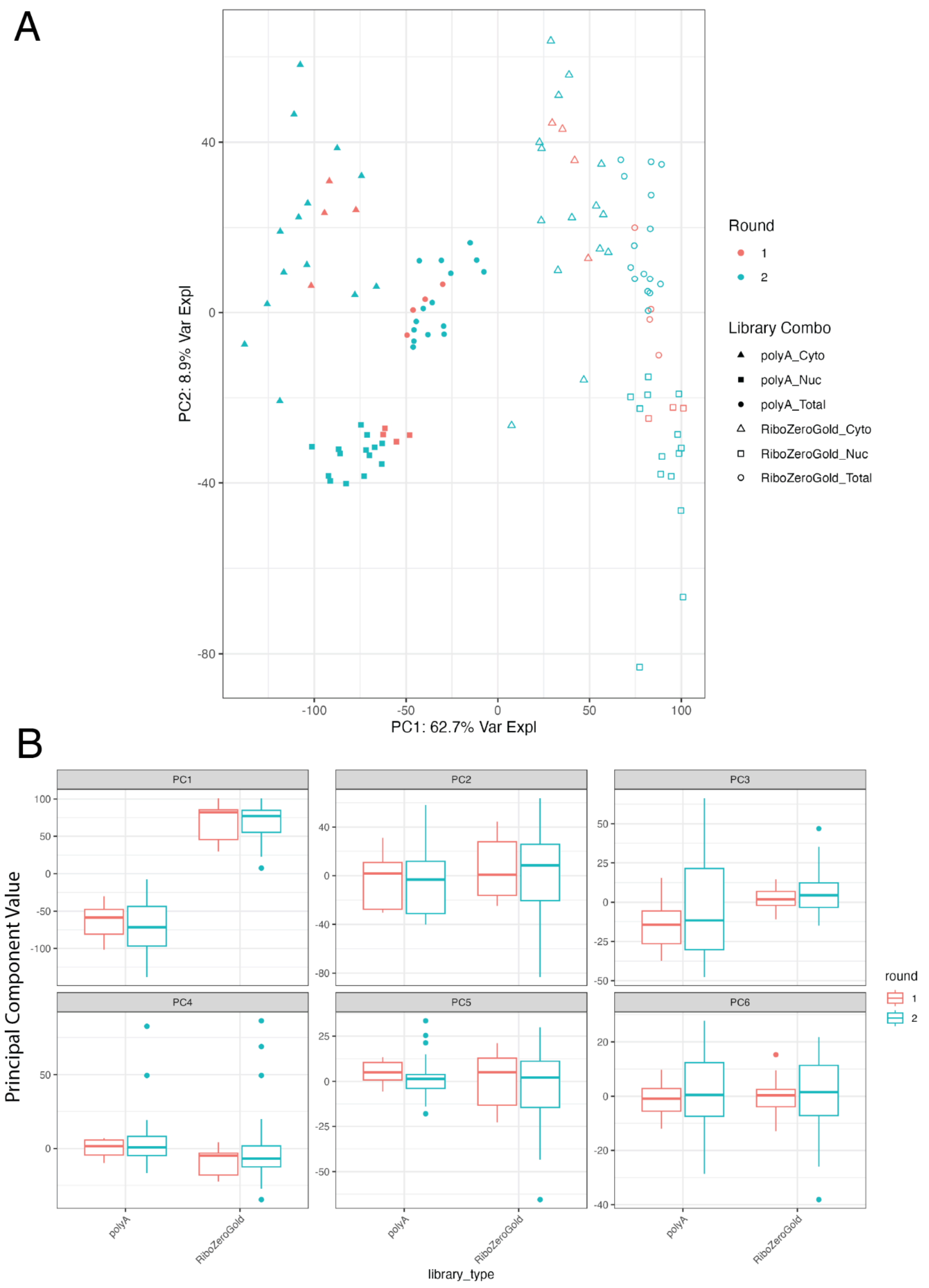
A
B
PC2: 8.9% Var Expl
PC1: 62.7% Var Expl
Round
1
2
Library Combo
polyA_Cyto
polyA_Nuc
polyA_Total
RiboZeroGold_Cyto
RiboZeroGold_Nuc
RiboZeroGold_Total
Principal Component Value
PC1
PC2
PC3
PC4
PC5
PC6
round
polyA
RiboZeroGold
library_type

Fig. S4: Bulk RNA-seq data Principal Component Analysis (PCA). A. PC1 versus 2 colored by sequencing round, point shape shows the library type and RNA extraction method **B.** Boxplots of the first six principal components (y-axis) by library type (x-axis) and sequencing round (color).

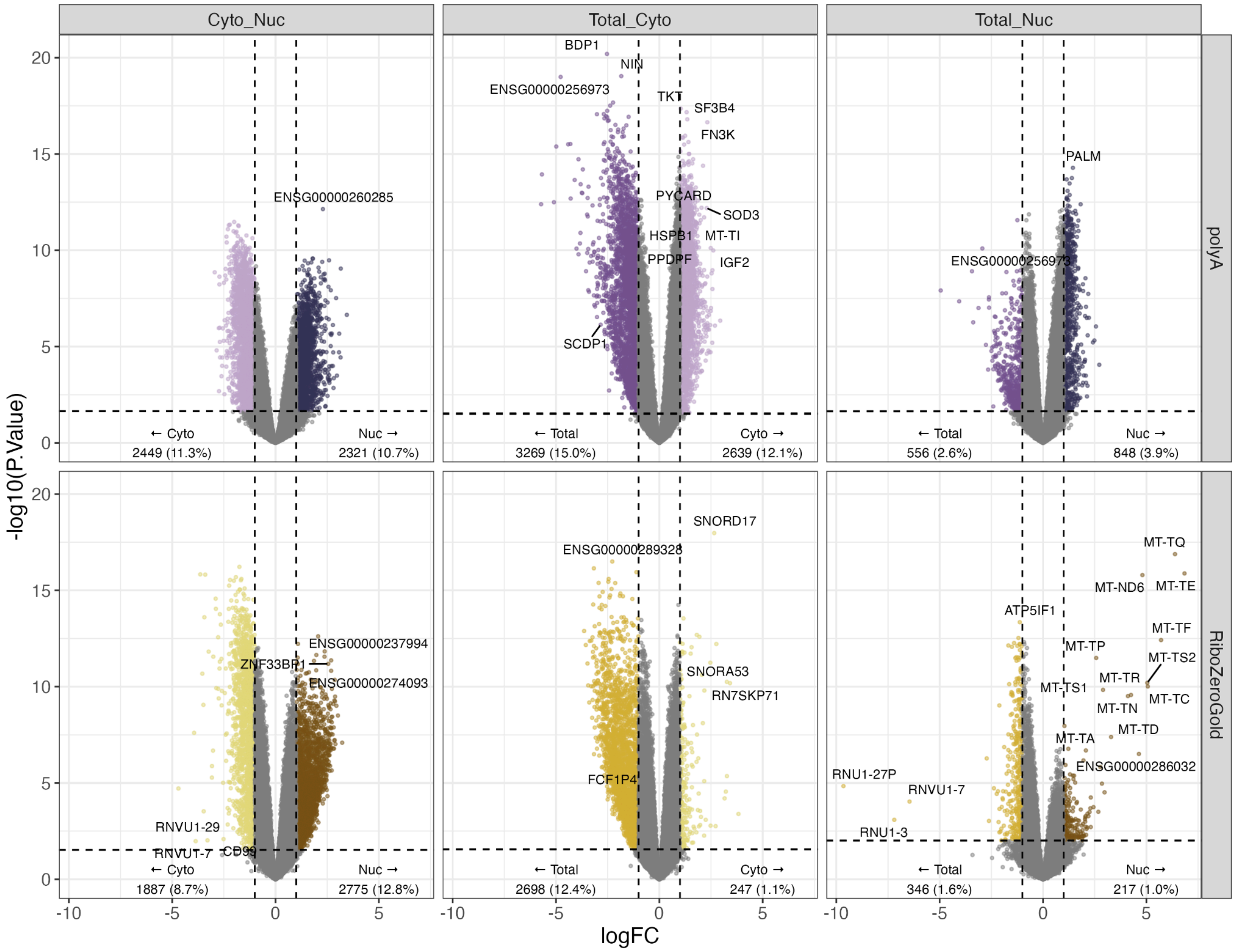

Fig. S5: Volcano plots for RNA extraction Differential Gene Expression analysis. Samples were separated by library types (rows) and the RNA extractions: cytosolic (Cyto, light color), total cell (Total, intermediate color), or nuclear (Nuc, dark color) samples were compared by differential expression in a pair-wise fashion (columns). Related to **Figure 1E**.

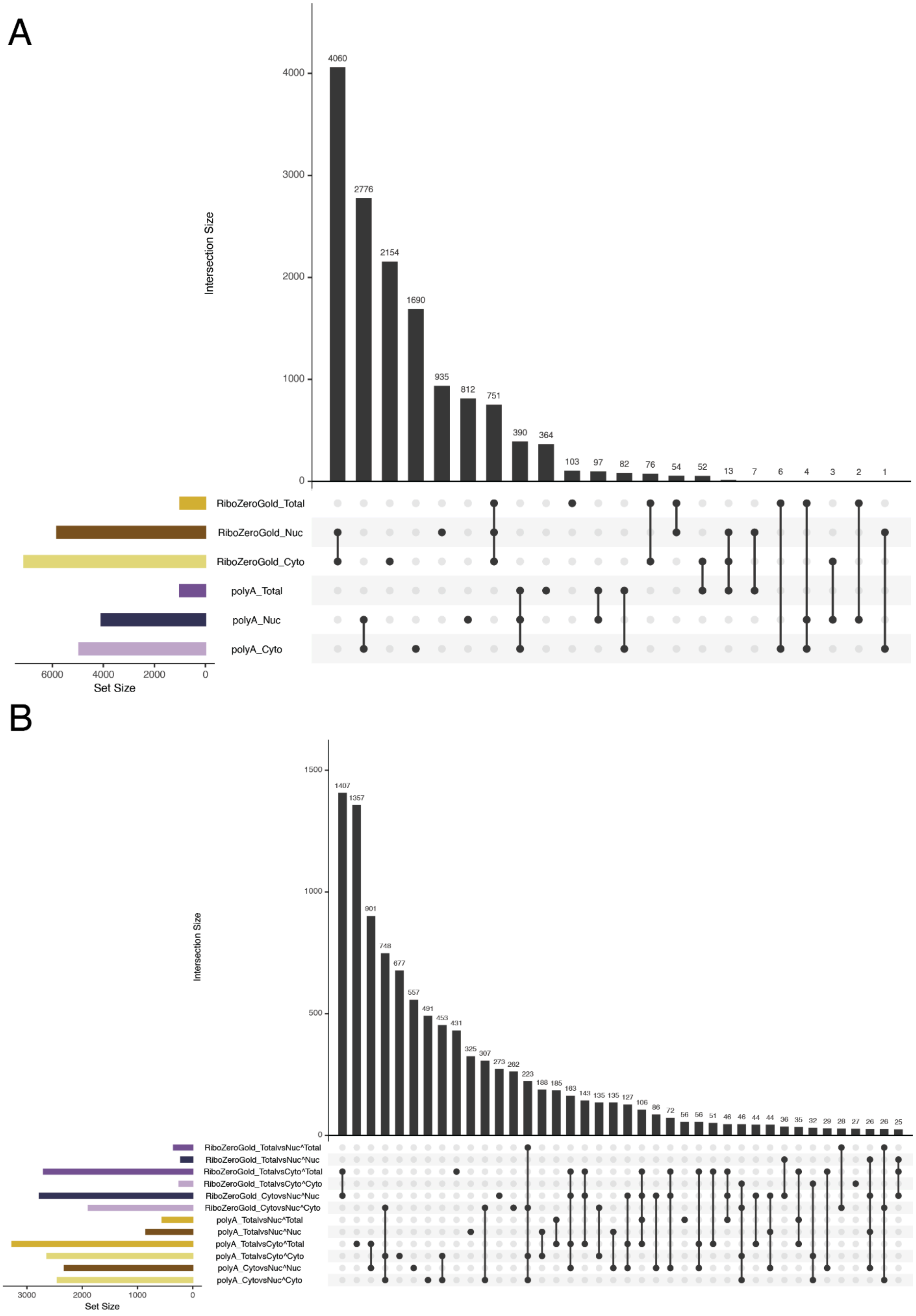
A
Intersection Size
4000
3000
2000
1000
0
4060
2776
2154
1690
935
812
751
390
364
103
97
82
76
54
52
13
7
6
4
3
2
1
RiboZeroGold_Total
RiboZeroGold_Nuc
RiboZeroGold_Cyto
polyA_Total
polyA_Nuc
polyA_Cyto
6000
4000
2000
0
Set Size
B
Intersection Size
1500
1000
500
0
1407
1357
901
748
677
557
491
453
431
325
307
273
262
223
188
185
163
143
135
135
127
106
88
72
56
56
51
46
46
44
44
36
35
32
29
28
27
26
26
25
RiboZeroGold_TotalvsNuc^Total
RiboZeroGold_TotalvsNuc^Nuc
RiboZeroGold_TotalvsCyto^Total
RiboZeroGold_TotalvsCyto^Cyto
RiboZeroGold_CytovsNuc^Nuc
RiboZeroGold_CytovsNuc^Cyto
polyA_TotalvsNuc^Total
polyA_TotalvsNuc^Nuc
polyA_TotalvsCyto^Total
polyA_TotalvsCyto^Cyto
polyA_CytovsNuc^Nuc
polyA_CytovsNuc^Cyto
3000
2000
1000
0
Set Size

Fig. S6: Upset plots for Differentially Quantified Genes. Displays the overlap between sets of differentially quantified genes for tests between **A.** library type (ex. RiboZeroGold_Total vs. polyA_Total) or **B.** RNA extraction (ex. RiboZeroGold_Total vs. RiboZeroGold_Cyto, where over quantified genes in Total would be notated as RiboZeroGold_TotalvsCyto^Total). Left barplots are colored by the combination of the library preparation and RNA extraction the set of genes are over quantified in. Related to **Figure 1E**, **Additional file 1: Fig. S5**.

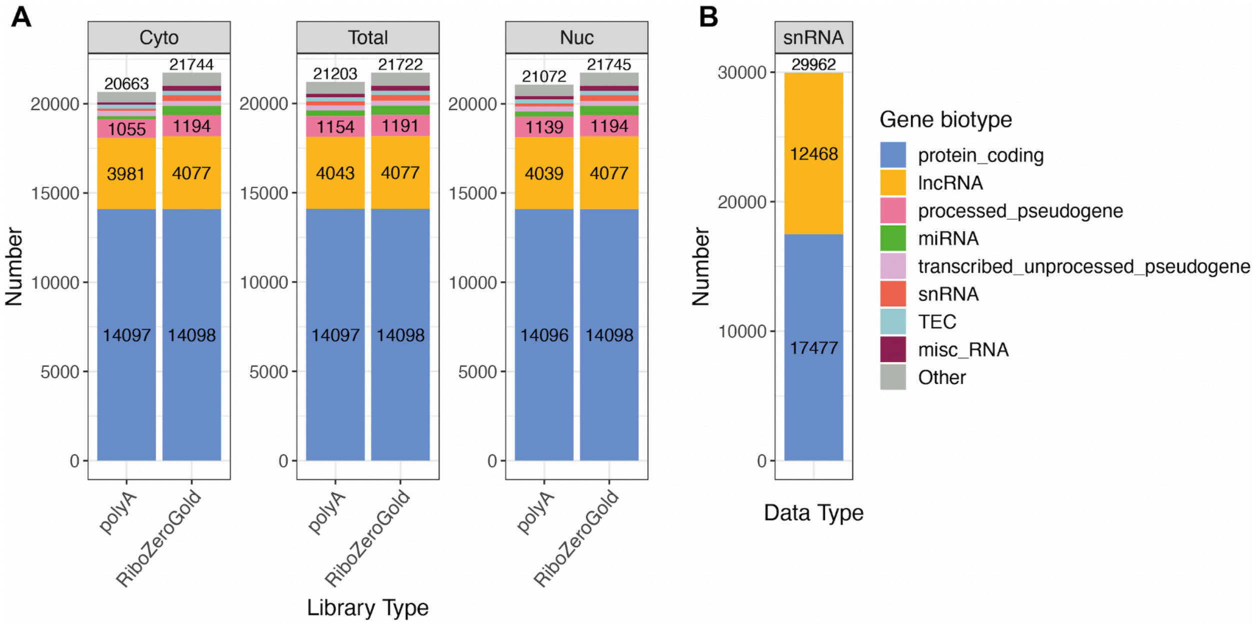

Fig. S7: Biotypes of expressed genes in bulk and snRNA-seq datasets. The total number of expressed genes (after removing lowly expressed genes) and their biotypes in **A**. Each of the 6 bulk RNA-seq libraries, comparing polyA vs RiboZeroGold in cytoplasmic, total, and nuclear RNA samples, and in **B**. snRNA-seq samples. These are the genes used as input for the DQG analysis. Related to **Figure 1**.

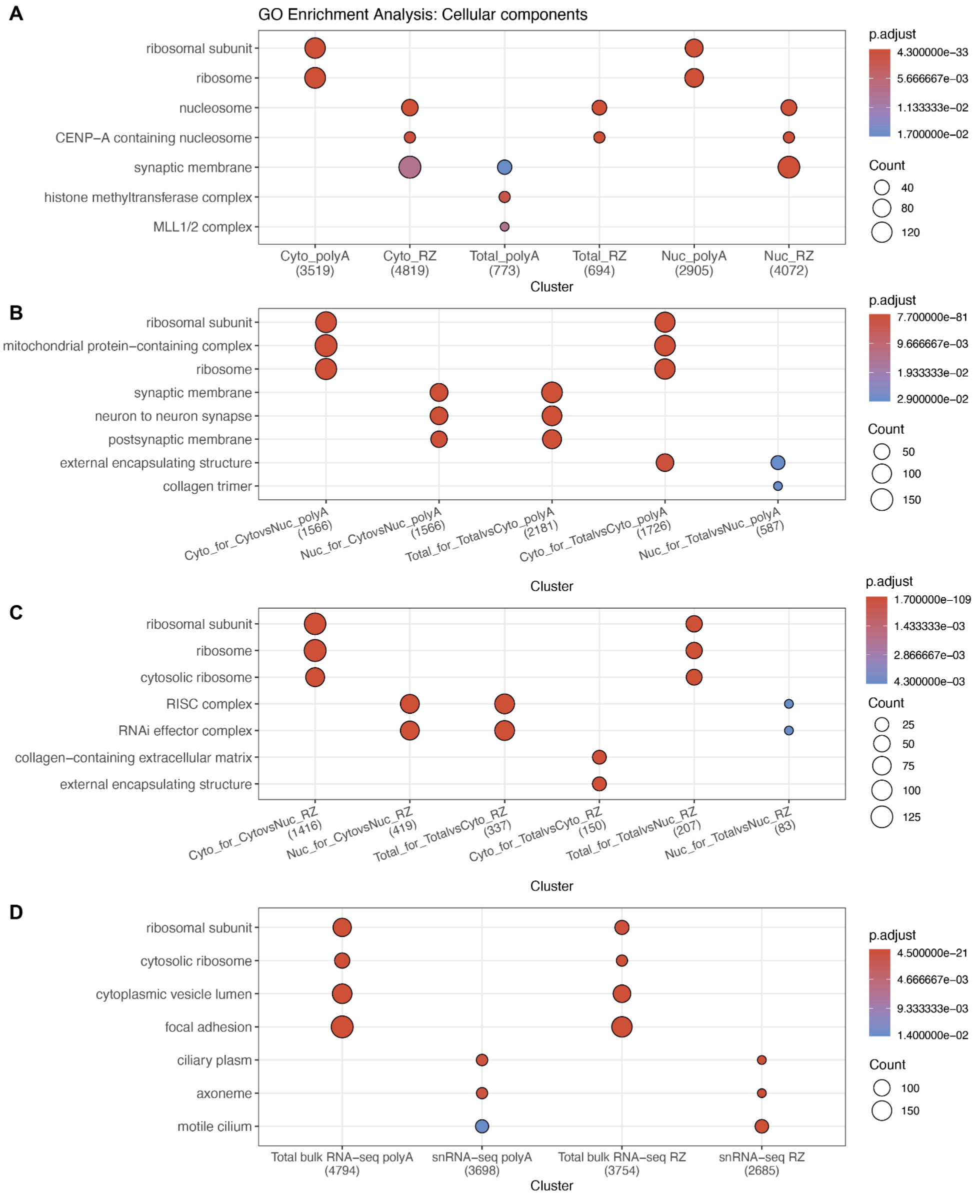

Fig. S8: Enrichment of gene ontology cellular component terms in DQGs. Comparison of gene sets defined by either (1; Y-axis) having common cellular components (CC) in the Gene Ontology (GO) knowledgebase, or (2; X-axis) being significantly differentially quantified (FDR<0.05) between **A**. library types in the same RNA fractions, RNA extractions in the same library types for **B**. polyA and **C**. RiboZeroGold, respectively, and **D**. sequencing assay types (snRNA-seq compared against Total RNA-seq). Gene clusters without significant enrichments are excluded. The numbers below each DQG group (X-axis) correspond to the number of genes in each group that are also annotated in the GO knowledgebase. Count is the size of the overlap between genes annotated in each CC GO term and the DQG groups. Only the top 2 most significant enriched CC GO terms per DQG group are shown; some of the CC GO terms overlap. Related to **Figure 1E-F**, **Additional file 1: Fig. S5**.

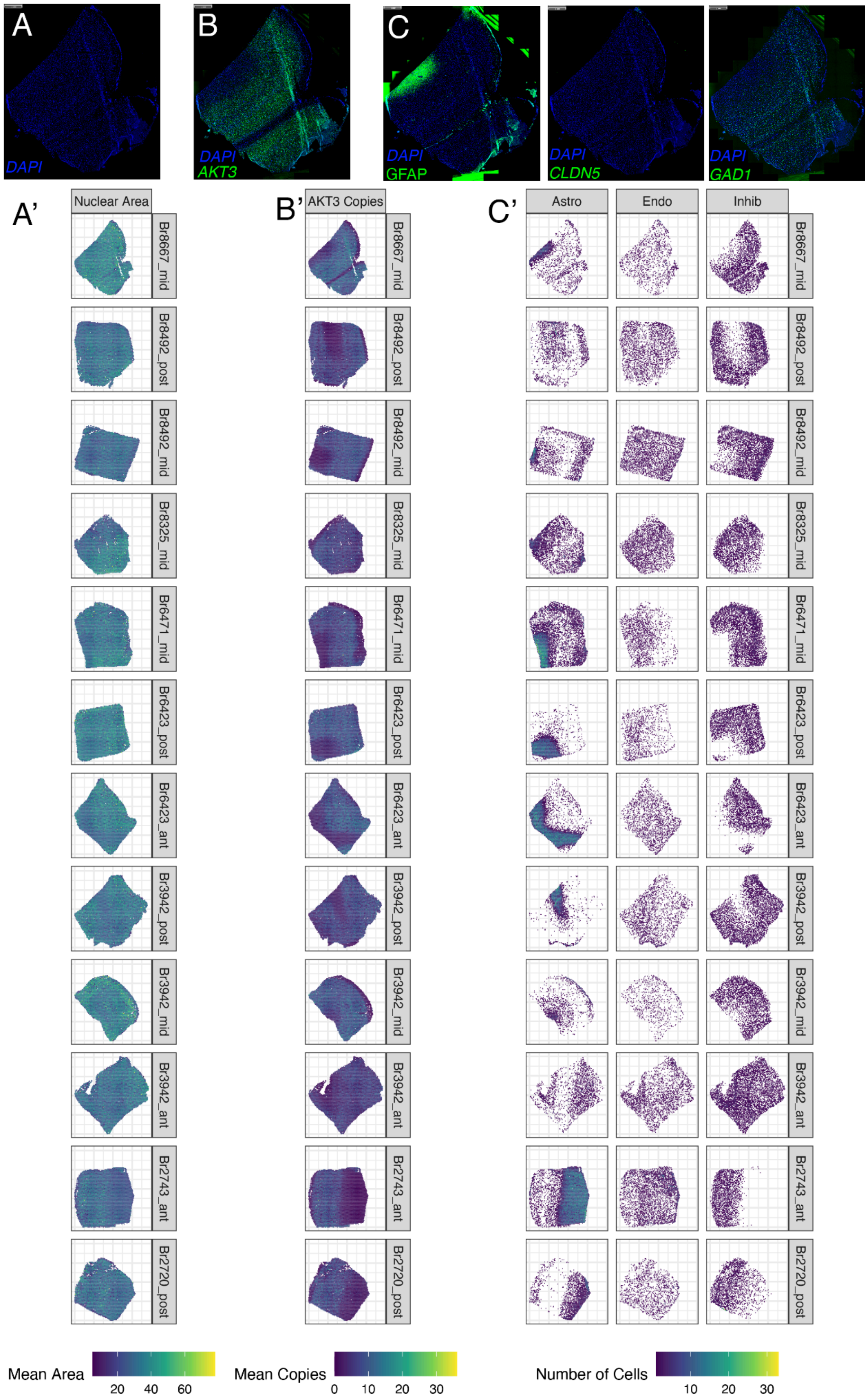
A
DAPI
B
DAPI
AKT3
C
DAPI
GFAP
DAPI
CLDN5
DAPI
GAD1
A'
Nuclear Area
B'
AKT3 Copies
C'
Astro
Endo
Inhib
Br8667_mid
Br8492_post
Br8492_mid
Br8325_mid
Br6471_mid
Br6423_post
Br6423_ant
Br3942_post
Br3942_mid
Br3942_ant
Br2743_ant
Br2720_post
Mean Area
20
40
60
Mean Copies
0
10
20
30
Number of Cells
10
20
30

Fig. S9: Representative fluorescence images and corresponding hex plots of all RNAScope/IF circle combination samples. Raw fluorescence for representative sample Br8667_mid: **A.** nuclear DAPI signals, **B.** DAPI and *AKT3*, **C.** DAPI and cell type probes/antibodies GFAP, CLDN5, and *GAD1*. Hex plots (`bins = 100`) from all circle samples summarizing: **A'.** mean nuclear area, **B'.** mean copies of *AKT3*, and **C'.** number of cells from the tagged cell types (Astro, Endo, and Inhib). Related to **Figure 2**.

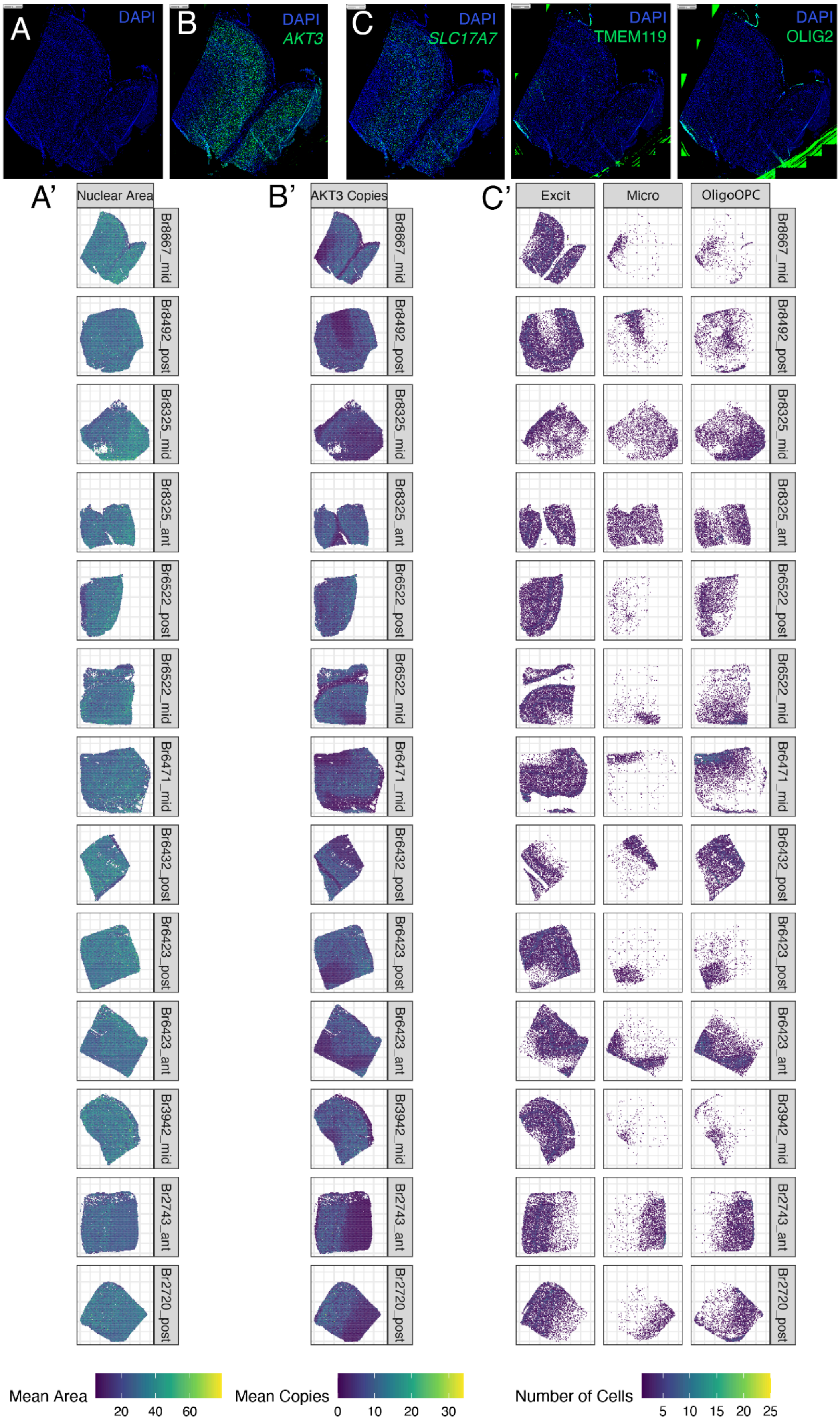
A
DAPI
B
DAPI
AKT3
C
DAPI
SLC17A7
DAPI
TMEM119
DAPI
OLIG2
A'
Nuclear Area
B'
AKT3 Copies
C'
Excit
Micro
OligoOPC
Br8667_mid
Br8492_post
Br8325_mid
Br8325_ant
Br6522_post
Br6522_mid
Br6471_mid
Br6432_post
Br6423_post
Br6423_ant
Br3942_mid
Br2743_ant
Br2720_post
Mean Area
20
40
60
Mean Copies
0
10
20
30
Number of Cells
5
10
15
20
25

Fig. S10: Representative fluorescence images and corresponding hex plots of all RNAScope/IF star combination samples. Raw fluorescence for representative sample Br8667_mid: **A.** nuclear DAPI signals, **B.** DAPI and *AKT3*, **C.** DAPI and cell type probes/antibodies *SLC17A7*, TMEM119, and OLIG2. Hex plots (`bins = 100`) from all circle samples summarizing: **A'.** mean nuclear area, **B'.** mean copies of *AKT3*, and **C'.** number of cells from the tagged cell types (Excit, Micro, and OligoOPC). Related to **Figure 2**.

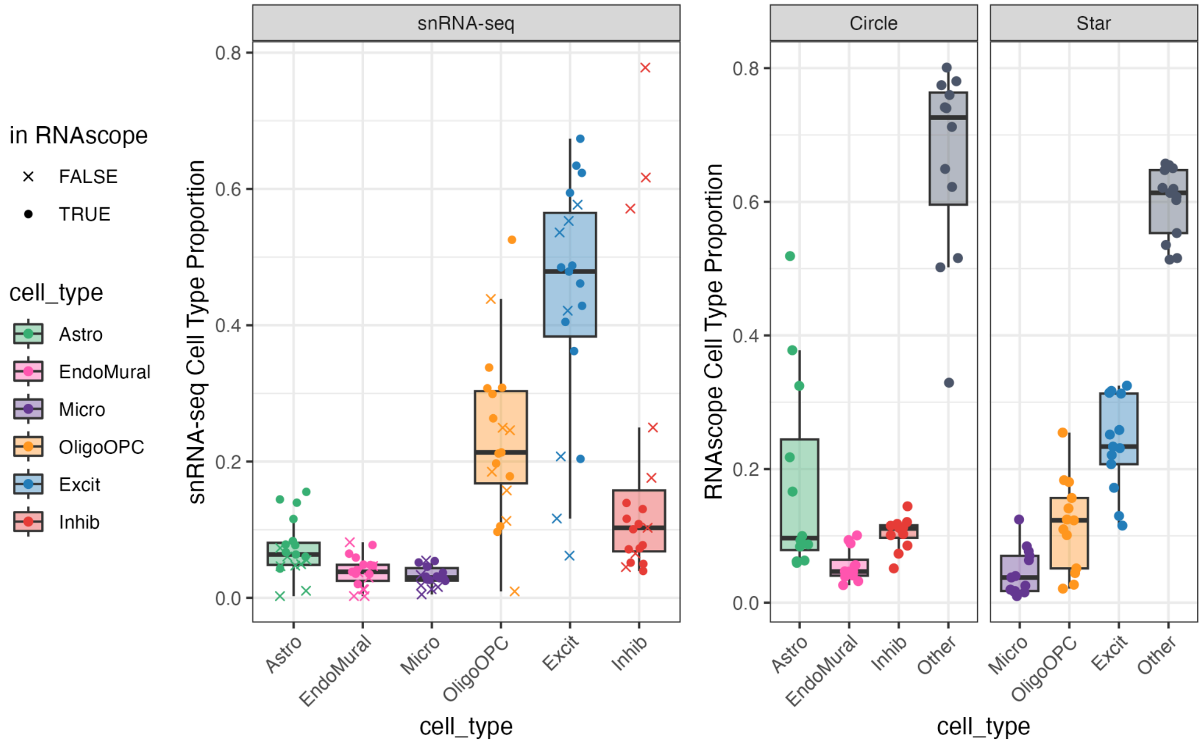

Fig. S11: Boxplots of cell type proportions calculated from snRNA-seq and RNAScope/IF data. (**Left Side**) This side shows the snRNA-seq cell type proportions at the broad cell type resolution for all 19 tissue blocks for which snRNA-seq data was previously generated [40]. A filled dot marks tissue blocks for which RNAScope/IF data was also generated and passed quality control checks. Those absent are labeled with an “x”. (**Right Side**) This side shows the RNAScope/IF broad cell type proportions derived from the RNAScope/IF experiments for the Circle and Star combination of cell type markers. Cell type identities on the RNAScope/IF images were assigned using HALO (Indica Labs). Related to **Figure 2**.

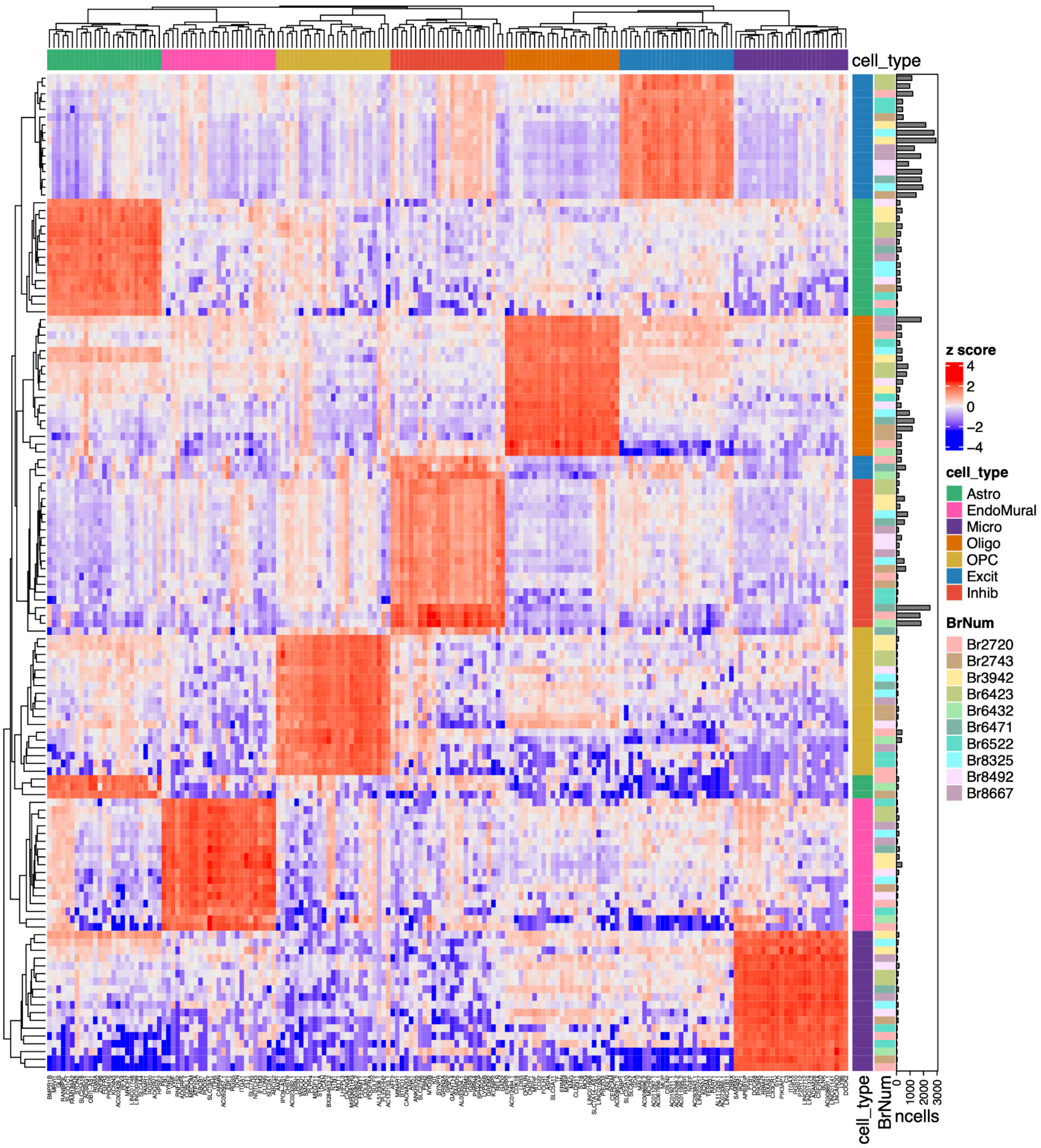

Fig. S12: Heatmap of the *Mean Ratio top25* marker genes for deconvolution. Normalized snRNA-seq counts (logcounts) were centered and scaled by gene to compute Z scores. Brain donor identifiers (`BrNum`) and cell types were used to annotate this heatmap made with *ComplexHeatmap* [74]. Genes are shown in the columns and nuclei on the rows, with the total number of nuclei (ncells) visualized as side barplots. Related to **Figure 3F**.

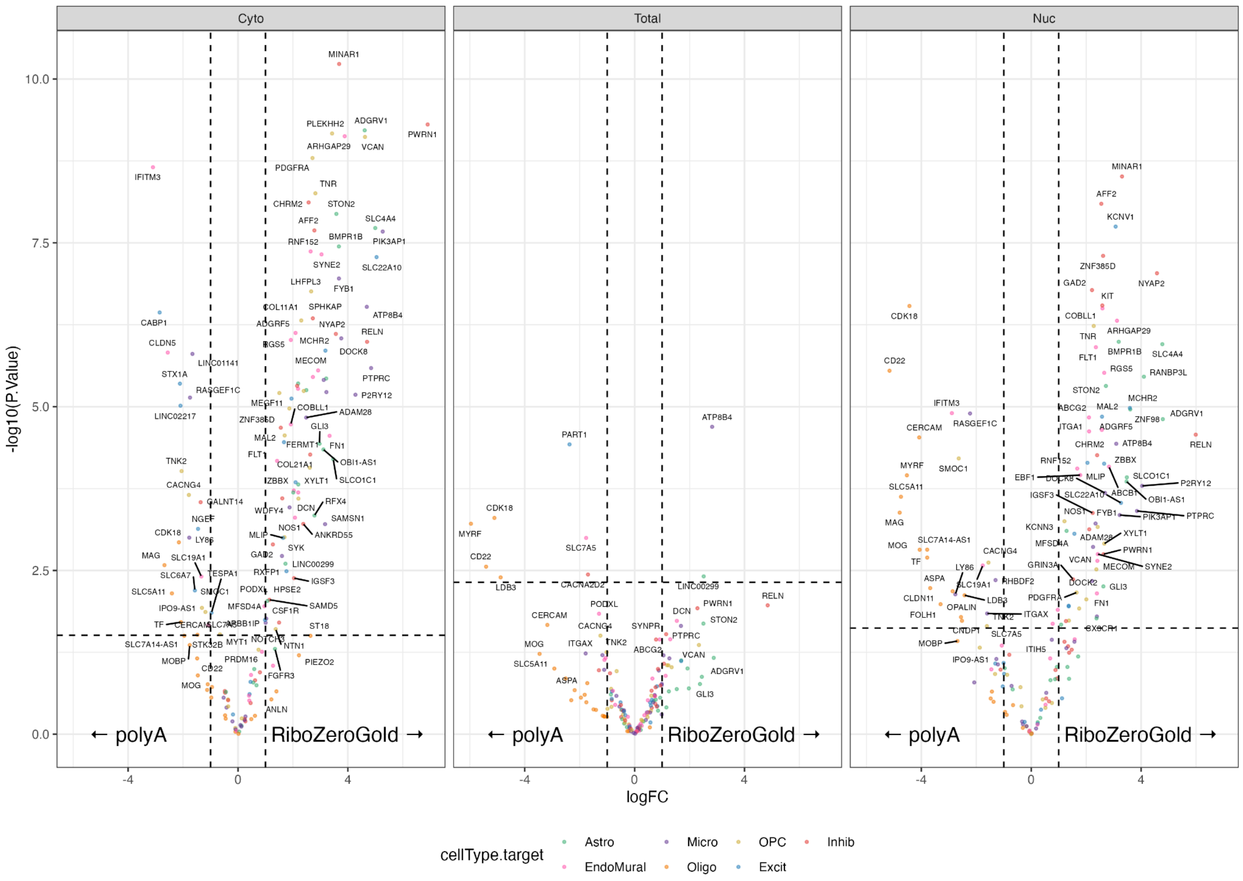

Fig. S13: Volcano plots for library type Differential Gene Expression analysis filtered to *Mean Ratio top25* marker genes. Plots are faceted by RNA extraction methods. Horizontal dotted line denotes FDR < 0.05 cutoff, vertical dotted lines are logFC = -1 and 1. Related to **Figure 1E** and **Figure 3**.

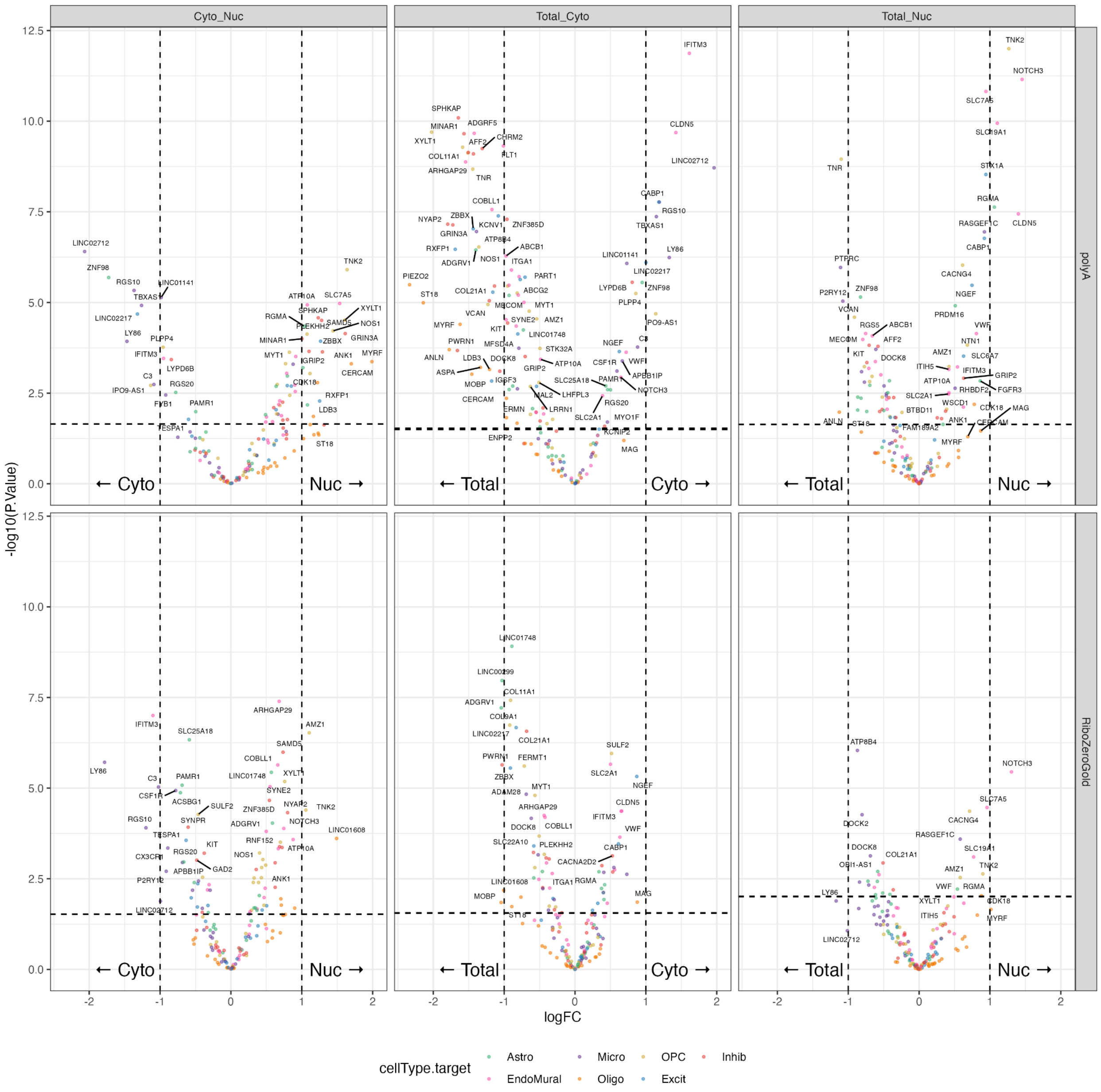

Fig. S14: Volcano plots for RNA extraction Differential Gene Expression analysis filtered to *Mean Ratio top25* marker genes. Plots are faceted by library preparation (rows), and pairwise comparisons (columns). Horizontal dotted line denotes FDR < 0.05 cutoff, vertical dotted lines are logFC = -1 and 1**.** Related to **Additional file 1: Fig. S5** and and **Figure 3**.

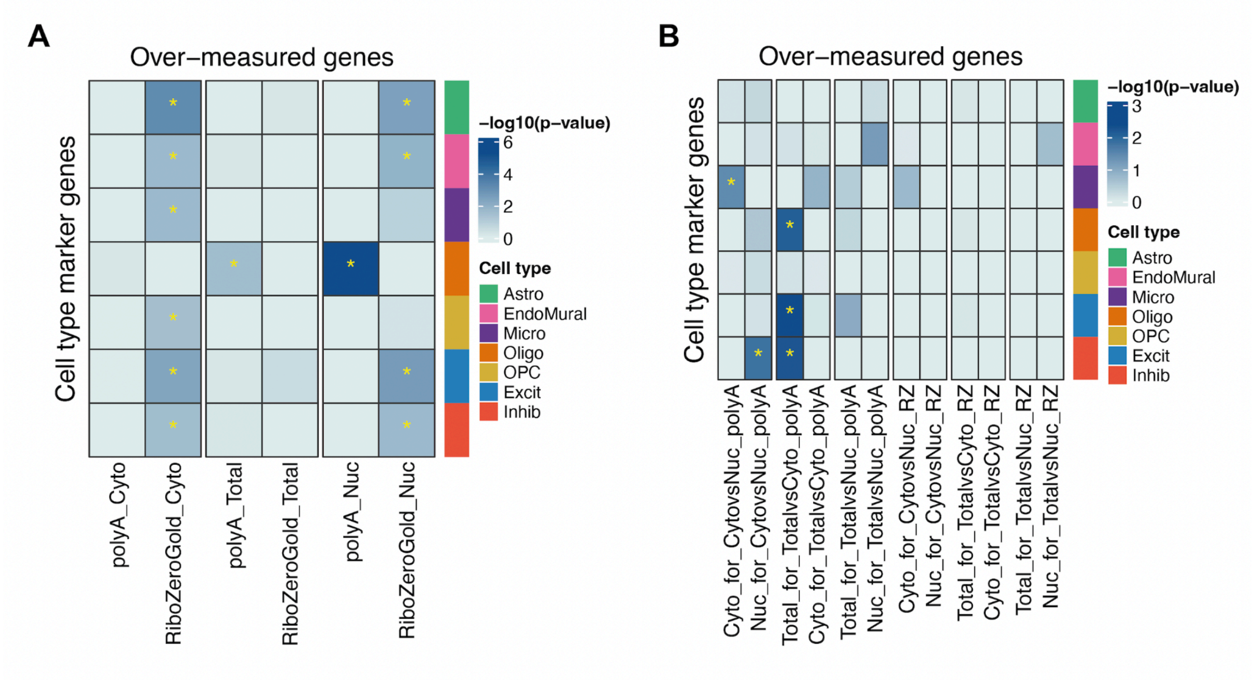

Fig. S15: Over-quantification of cell type marker genes in library type and RNA fraction RNA-seq libraries. The over-representation of top 25 *Mean Ratio* cell type marker genes among DQG groups between (**A**) polyA and RibZeroGold RNA library types, and (**B**) between Cyto, Nuc, and Total RNA extractions. Over-representation was assessed with one-sided Fisher's exact tests. The *p*-values for such enrichments are shown in the heat maps in a negative log10 scale. Significant associations (*p*-value <0.05) are indicated with a yellow "*". Related to **Figure 1E, Additional file 1: Fig. S5**, and **Figure 3**.

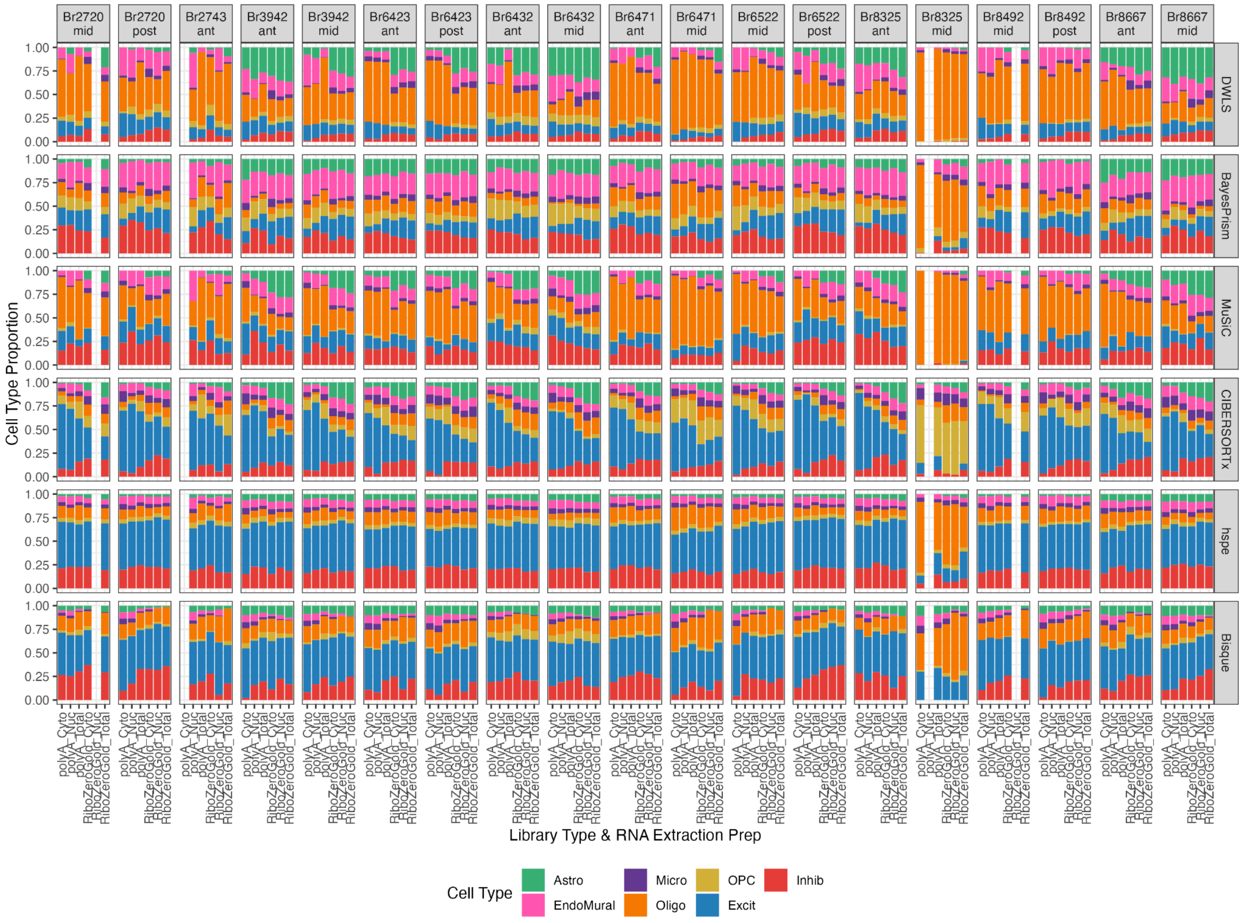

Fig. S16: Barplots of estimated cell type proportions from deconvolution methods. Each tissue block is a column, the x-axis categories are the six RNA extraction and library type combinations for the 110 bulk RNA-seq samples. The rows are the predictions from each of the six deconvolution methods. Columns are labeled by the tissue block, which is a combination of the brain donor identifier (`BrNum`) and the anterior-posterior axis location of the tissue block (anterior: ant, middle: mid, or posterior: post). Related to **Figure 4**.

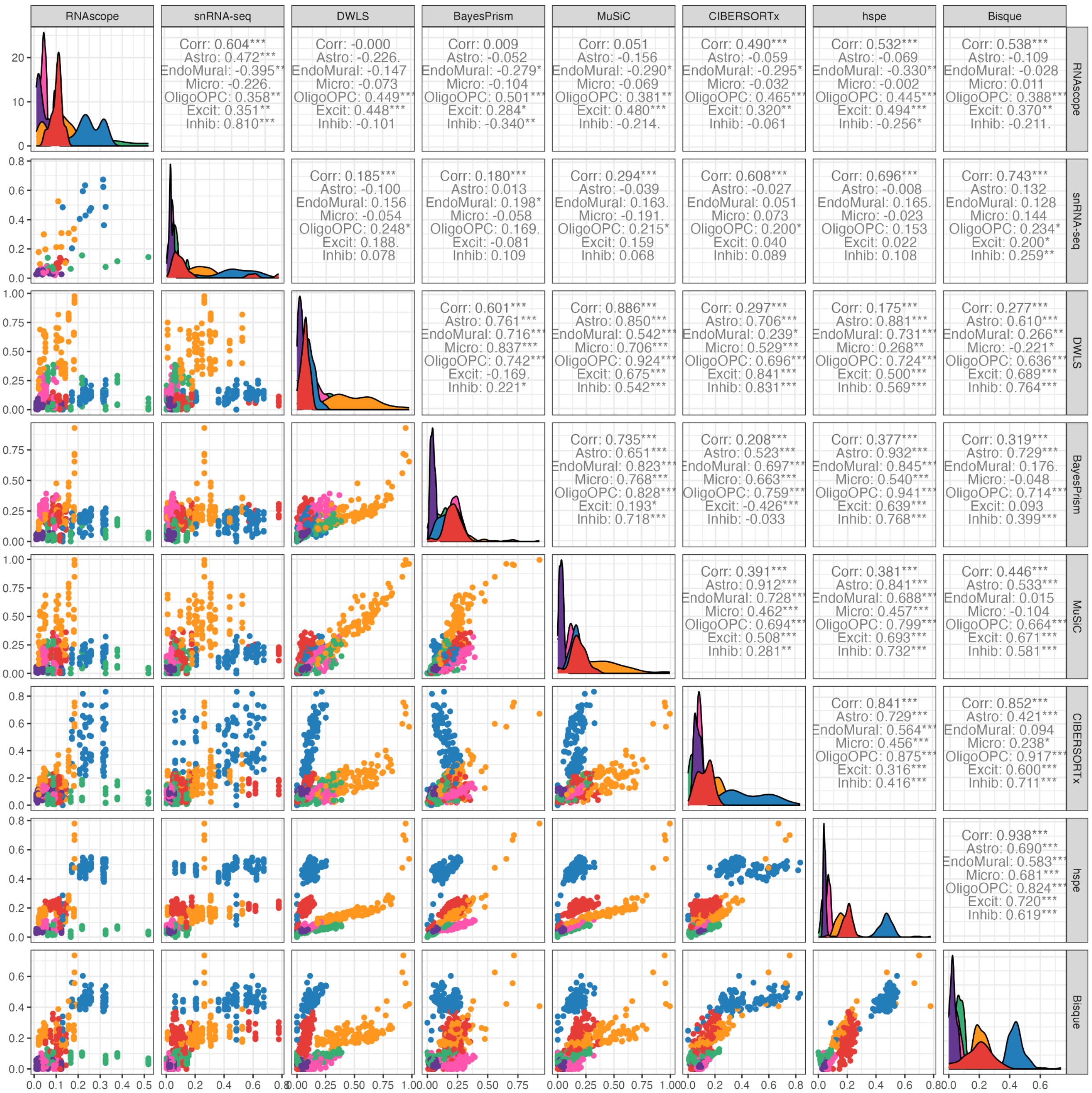

Fig. S17: Cell composition comparison for *Mean Ratio top25* results. Pairwise scatter plots of measured and estimated cell type proportions from the RNAScope/IF experiments, snRNA-seq data, and deconvolution methods using Mean Ratio top25 marker genes. Cell type proportions are colored by cell type and are shown in the lower triangle. Pearson correlation values (cor) calculated by `ggpairs()` from *GGally* [76] for each cell type are shown in the upper triangle. Density plots of the proportions are shown in the diagonal panels. Related to **Figure 4**.

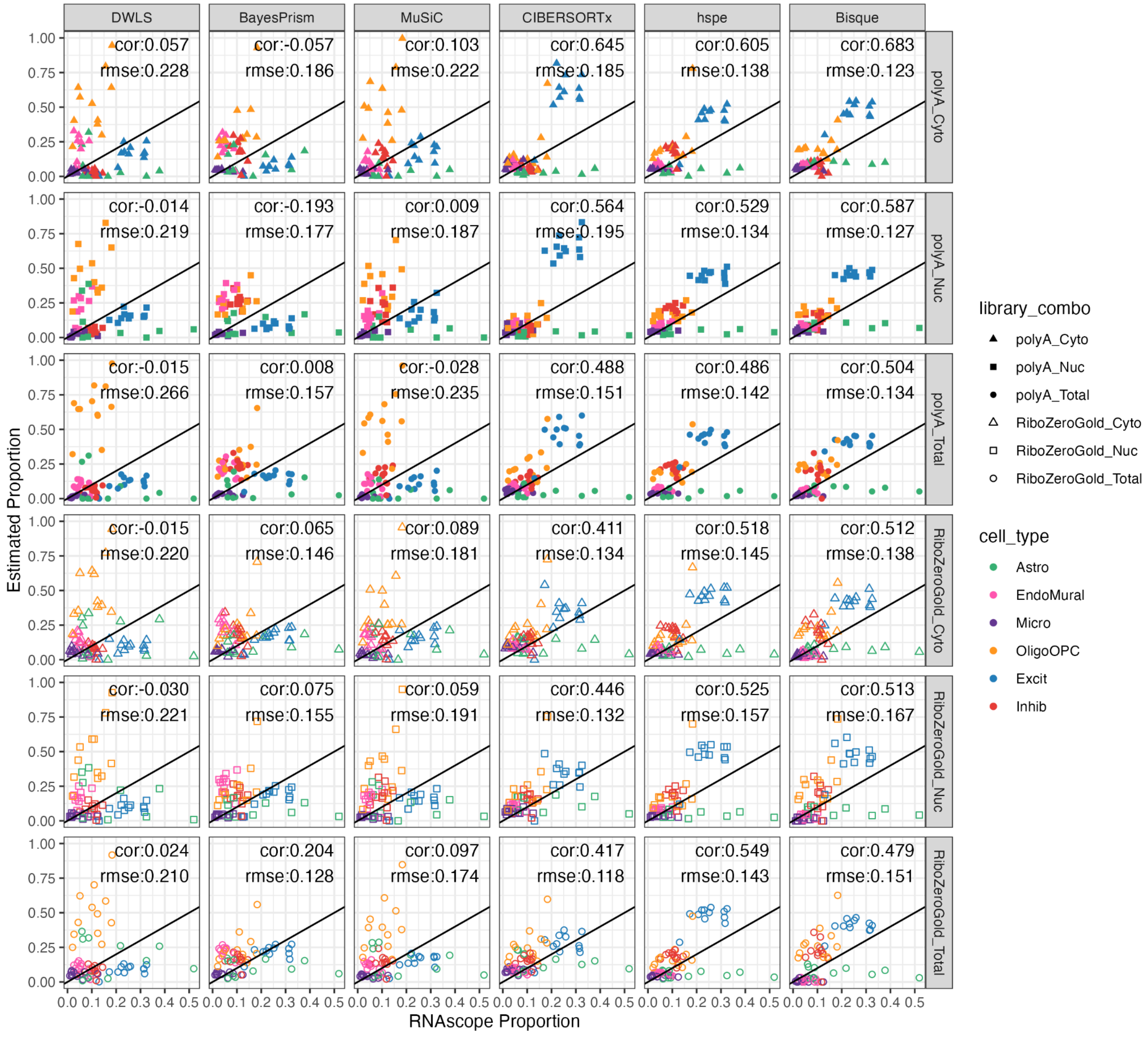

Fig. S18: Cell composition results against RNAScope/IF across bulk RNA library type and RNA extractions. Scatter plots of cell type proportions by RNA library combinations (columns) and deconvolution methods (rows). Points are colored by cell type and shaped by the RNA library combination. Pearson correlation (cor) and root mean squared error (rsme) values are shown for each panel. Related to **Figure 4**.

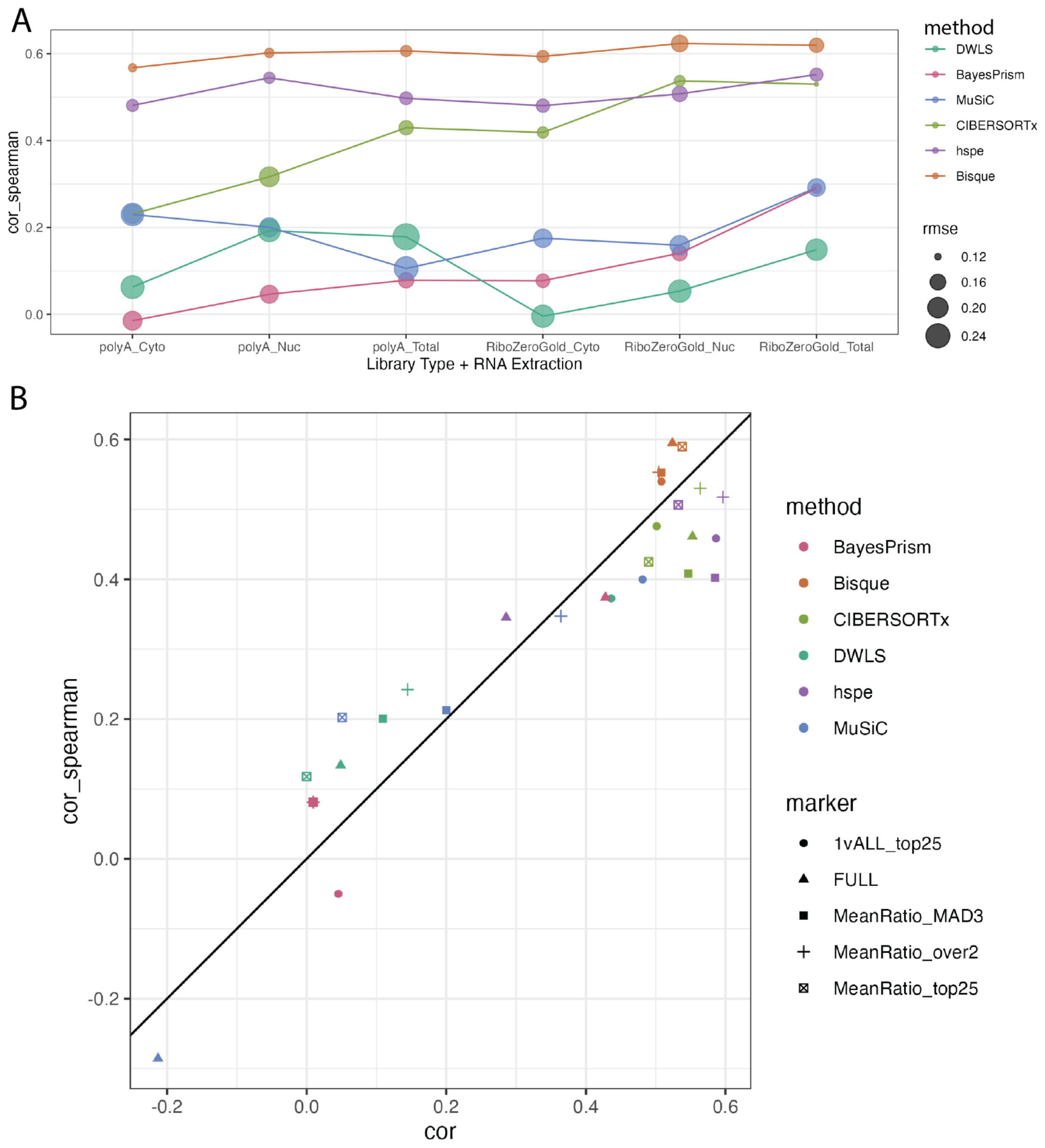

Fig. S19: Deconvolution method performance evaluated with Spearman Correlation. A. Spearman's correlation (cor_spearman) between the predicted proportions by deconvolution methods with MeanRatio_top25 marker genes and the estimated RNAScope/IF proportions across RNA extraction method and library type combinations, point size reflects the rmse value (related to **Figure 4B**). **B.** Scatter plot of overall pearson correlation (cor) compared to Spearman's correlations (cor_spearman) for each method (color) and set of marker genes (point shape).

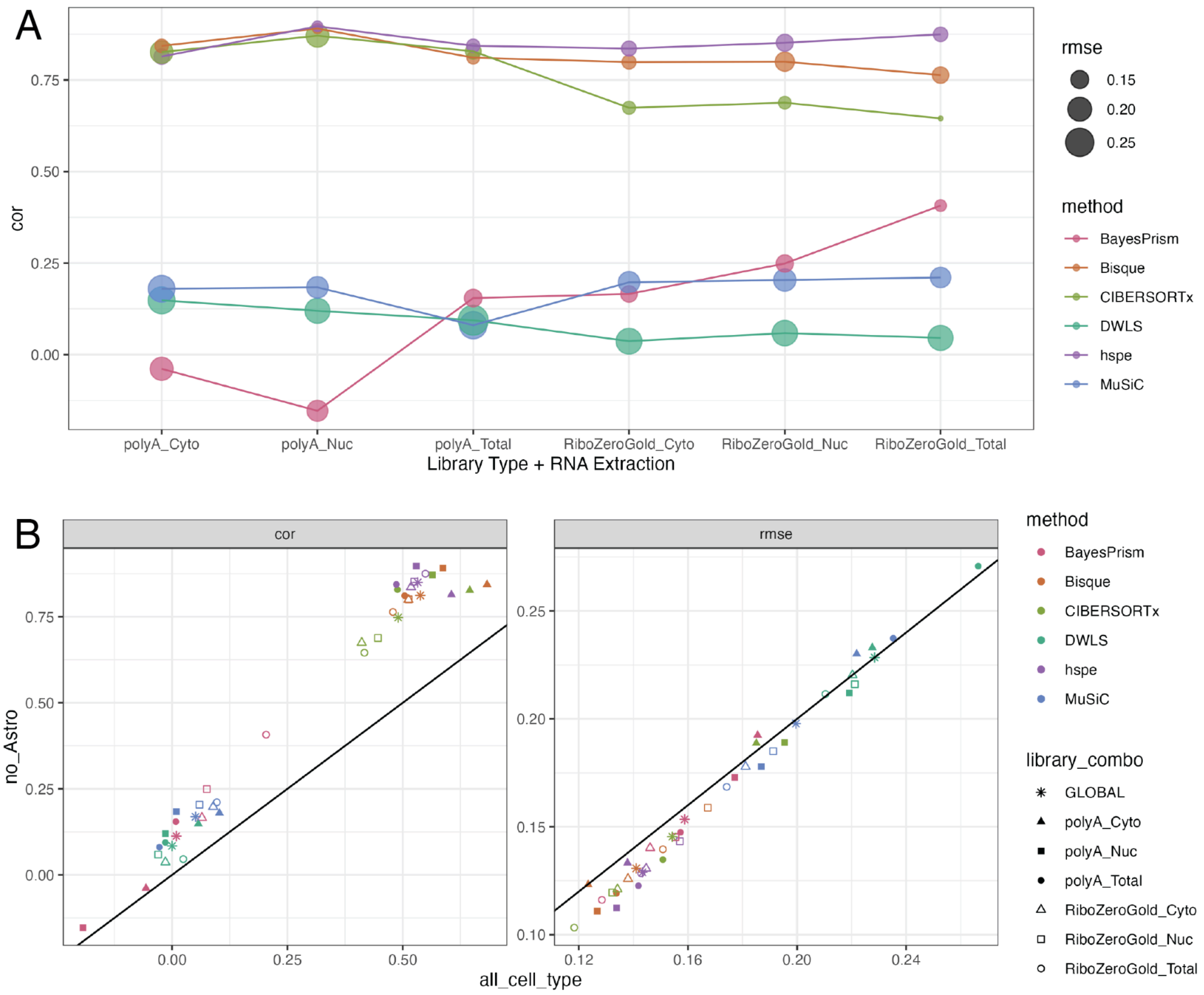

Fig. S20: Deconvolution method performance evaluated without Astro. A. Pearson correlation (cor) between the predicted proportions by deconvolution methods with MeanRatio_top25 marker genes and the estimated RNAScope/IF proportions across RNA extraction method and library type combinations, excluding Astro predictions, point size reflects the rmse value (related to **Figure 4B**). **B.** Scatter plot of pearson correlation (cor) and RMSE with all cell types and without Astro, across all samples (GLOBAL) and across RNA extraction method and library type combinations (point shape).

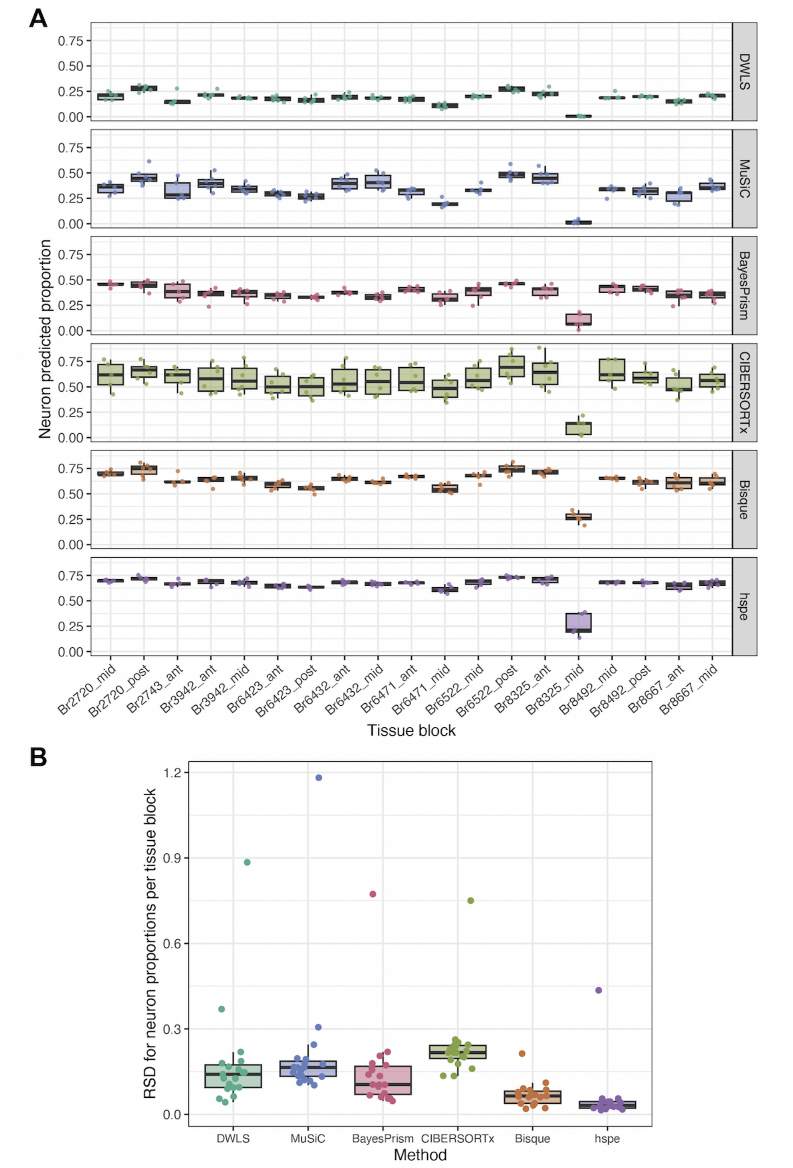

Fig. S21: Variation in estimated neuron proportions across bulk RNA-seq samples from each tissue block. A. Boxplots for the proportion of inhibitory and excitatory neurons (neuron predicted proportion) estimated by each deconvolution method across the six combinations of RNA-seq library preparation types and RNA extraction. **B**. Boxplots of relative standard deviation (RSD, also known as coefficient of variation; RSD = CV = σ / μ) in each tissue block for the neuron predicted proportions by each deconvolution method. The high outlier value corresponds to the `Br8325_mid` tissue block RSD, for which more variable and lower neuron proportions were predicted by the methods (A). Related to **Figure 5** and **Additional file 1: Fig. S16**.

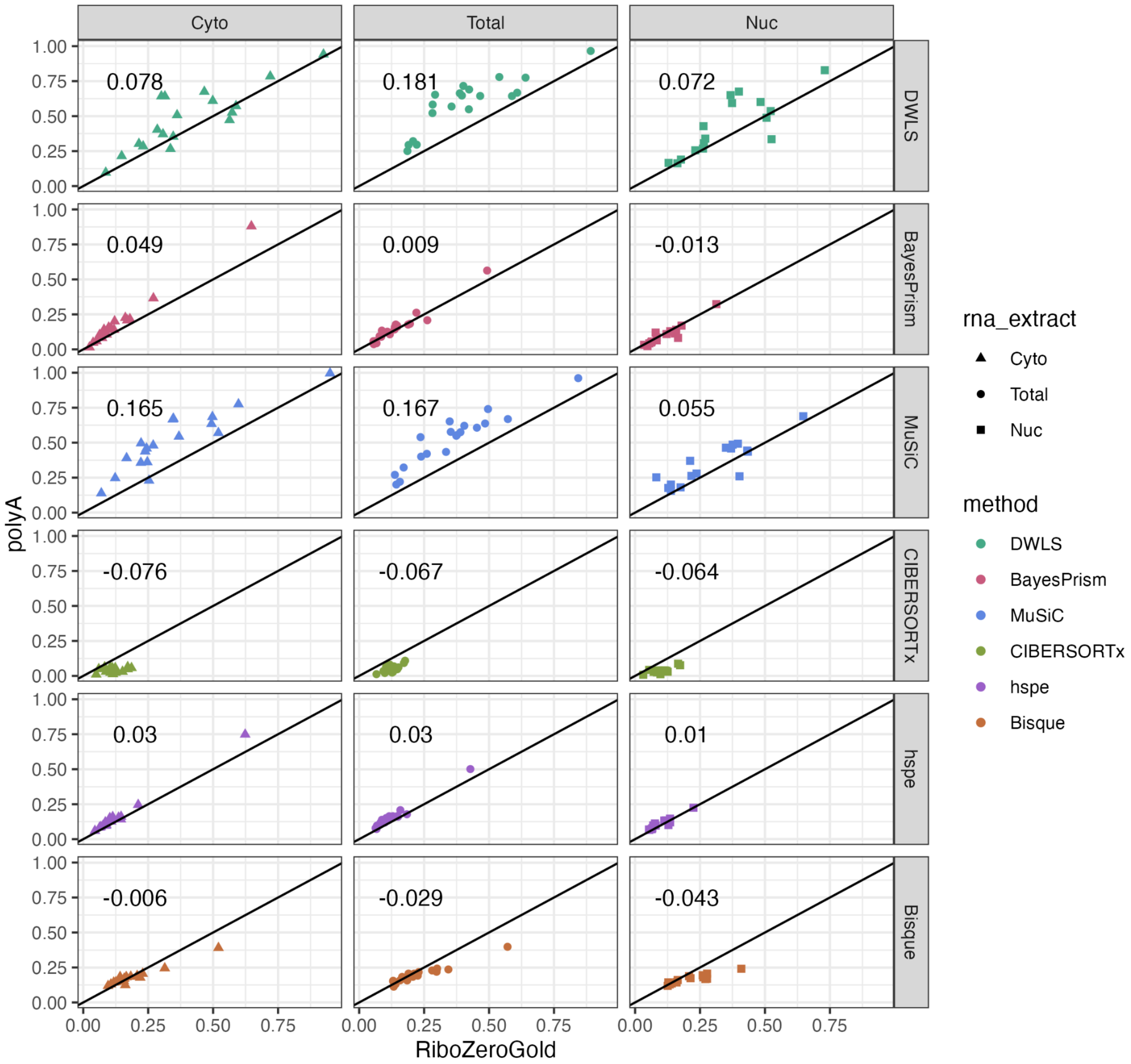

Fig. S22: Oligodendrocyte estimated proportion consistency across polyA and RiboZeroGold. Estimated oligodendrocyte proportion by the evaluated deconvolution methods (rows) using the *Mean Ratio top25* cell type marker genes as input. Proportions are compared between polyA and RiboZeroGold by RNA extraction (columns and shape). The $y = x$ line is shown as a black solid line. The mean difference (PolyA- RiboZeroGold) of estimated proportion Oligo is annotated in each plot. Related to **Figure 4**, **Additional file 1: Fig. S13**, **Additional file 1: Fig. S15**.

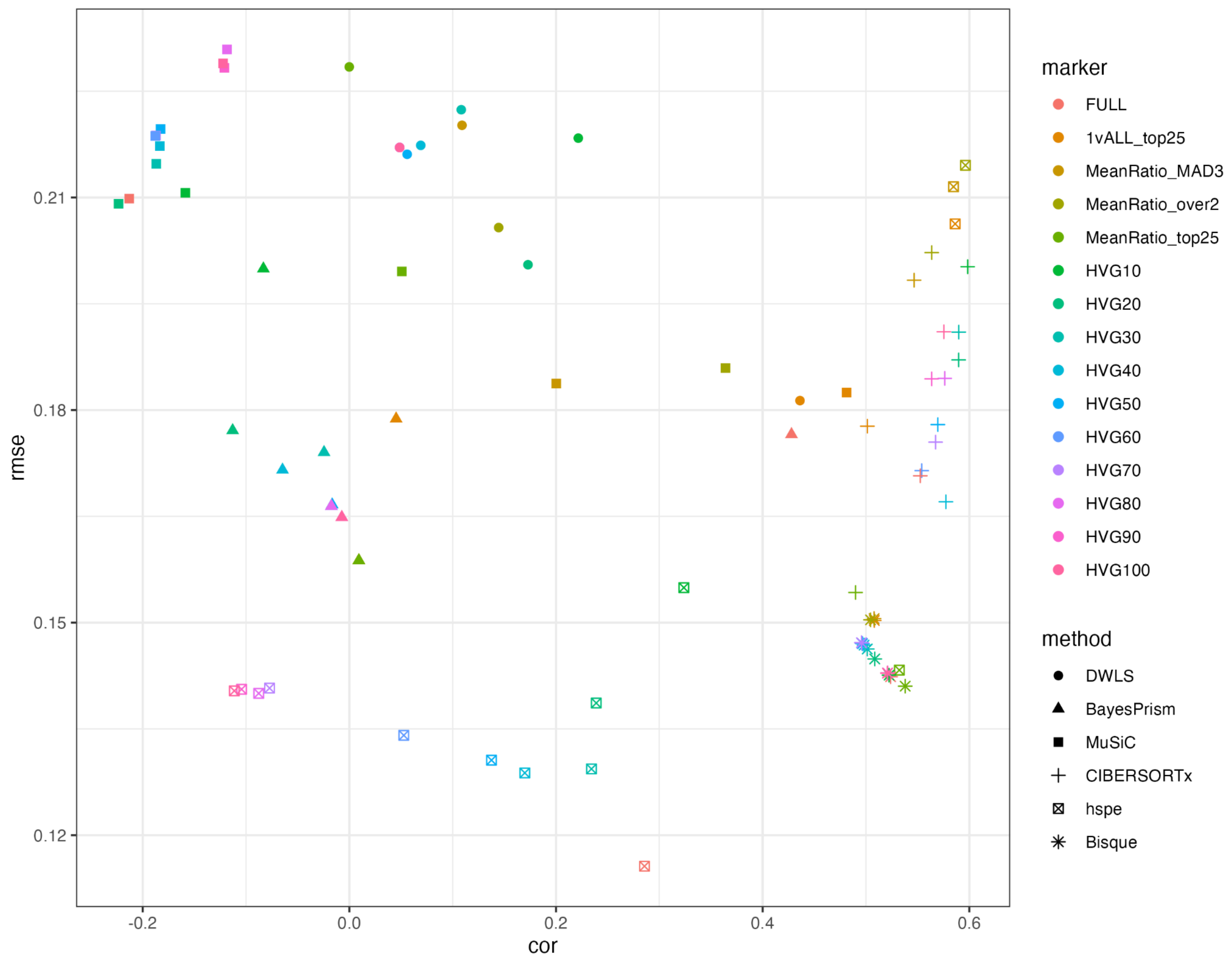

Fig. S23. Deconvolution method performance with various marker or gene sets. Scatter Plot between the overall Pearson's correlation (cor) and root mean squared error (rmse) values for cell type proportion predictions for each method (shape) and gene sets (point color) full set of common genes, marker gene sets, and 10-100% of highly variable genes (HVGs). Related to **Figure 5**.

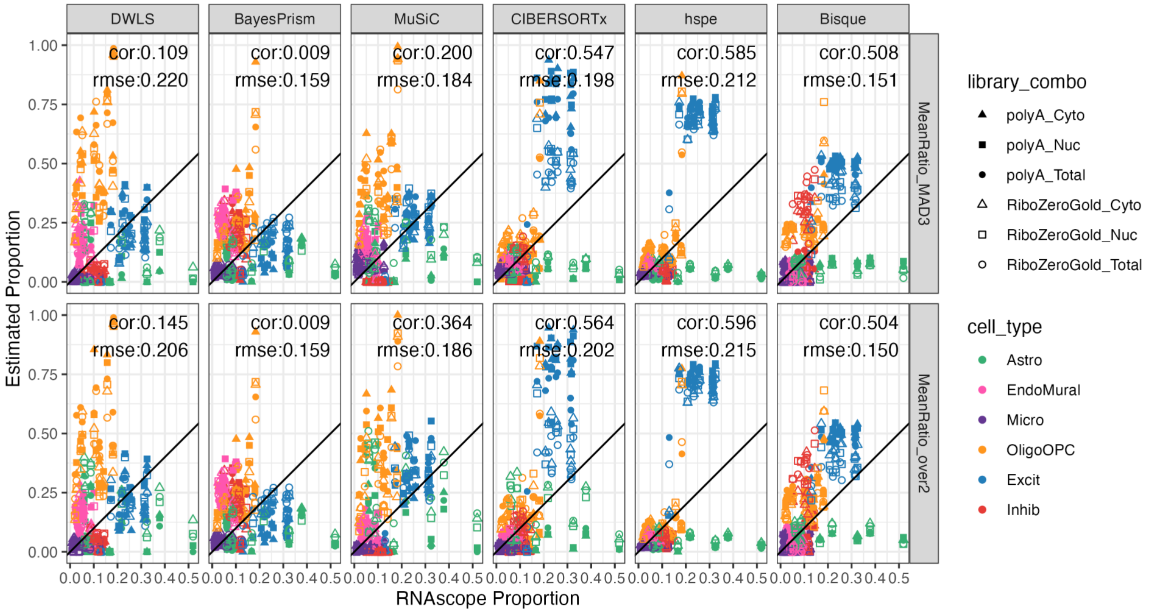

Fig. S24: Cell composition results *Mean Ratio* over 2 and MAD3. Scatter plot of cell type proportions estimated by RNAScope/IF (x-axis) vs. the predicted cell type proportions by the deconvolution methods for Mean Ratio over 2 and Mean ratio MAD3 marker sets. Points are colored by cell type and shaped by the combination of the bulk RNA-seq sample's library type and RNA extraction. Pearson correlation (cor) and root mean squared error (rsme) values are shown for each panel. Related to **Figure 5**.

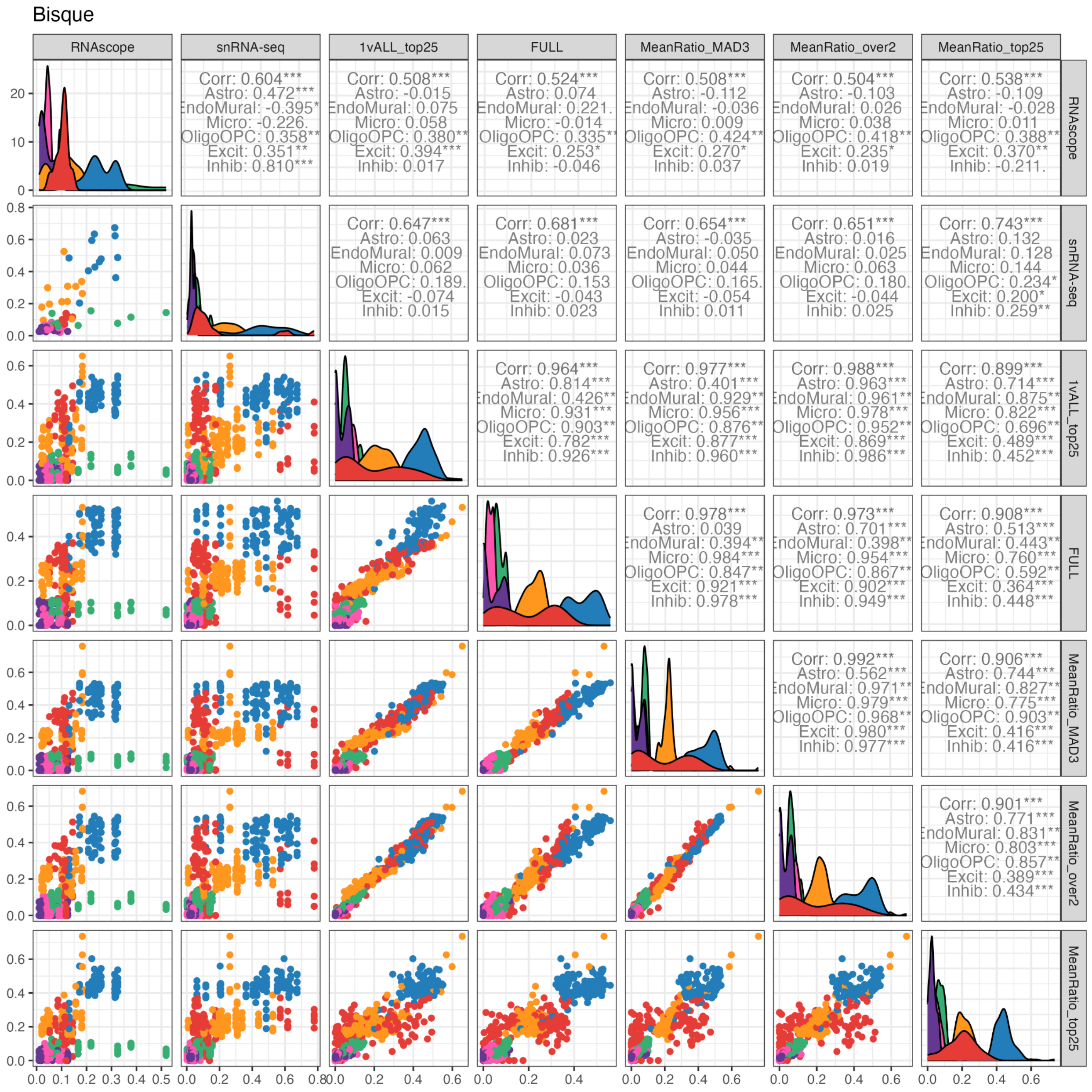

Fig. S25: Cell composition comparison for *Bisque* results across marker gene selection methods. Pairwise scatter plots of measured and estimated cell type proportions from the RNAScope/IF experiments, snRNA-seq data, and *Bisque* across five marker gene sets. Cell type proportions are colored by cell type and shown in the lower triangle. Pearson correlation values (cor) calculated by `ggpairs()` from *GGally* [76] for each cell type are shown in the upper triangle. Density plots of the proportions are shown in the diagonal panels. Related to **Figure 5**.

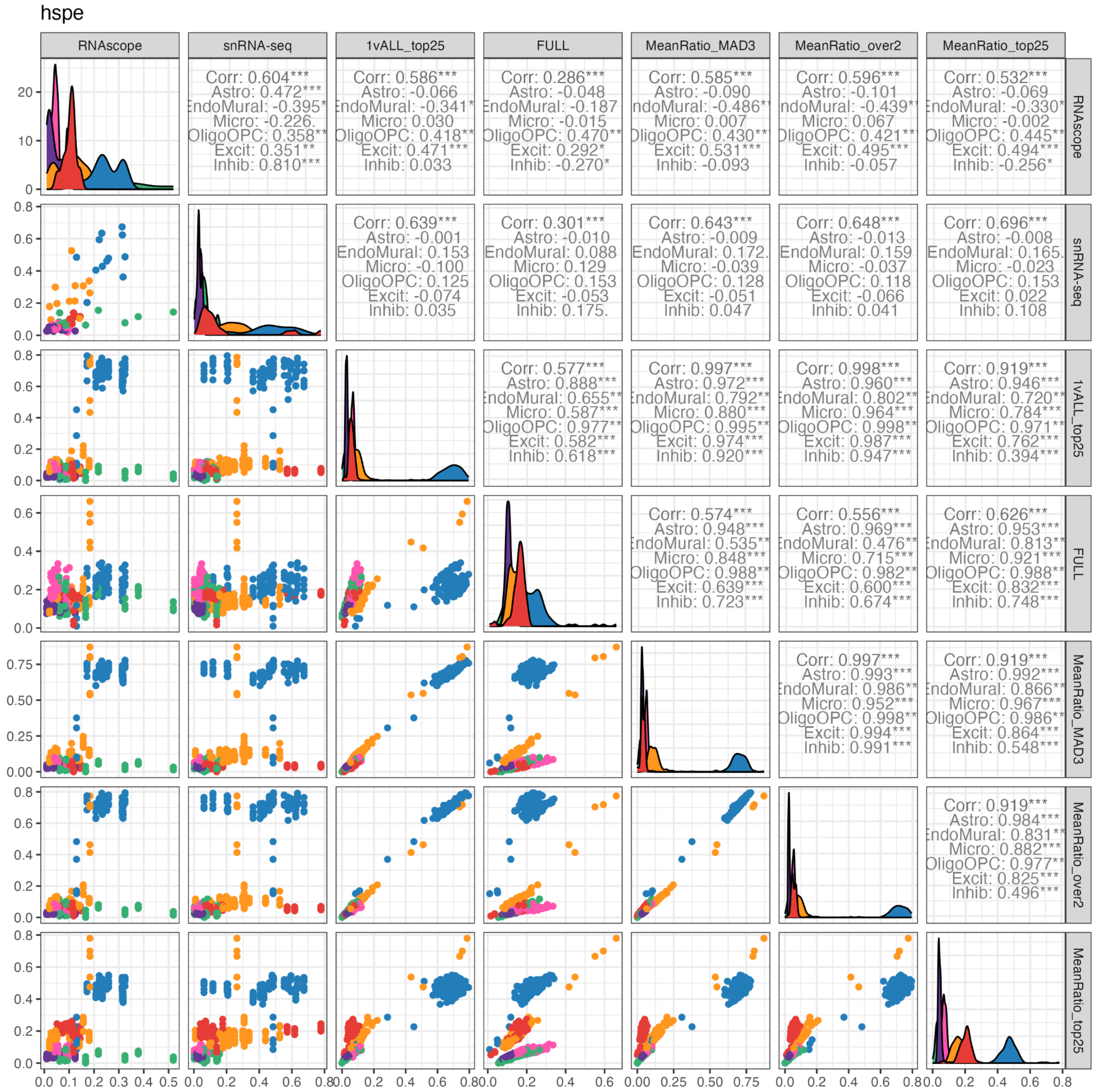

Fig. S26: Cell composition comparison for *hspe* results across marker gene selection methods. Pairwise scatter plots of measured and estimated cell type proportions from the RNAScope/IF experiments, snRNA-seq data, and *hspe* across five marker gene sets**.** Cell type proportions are colored by cell type and shown in the lower triangle. Pearson correlation values (cor) calculated by `ggpairs()` from *GGally* [76] for each cell type are shown in the upper triangle. Density plots of the proportions are shown in the diagonal panels. Related to **Figure 5**.

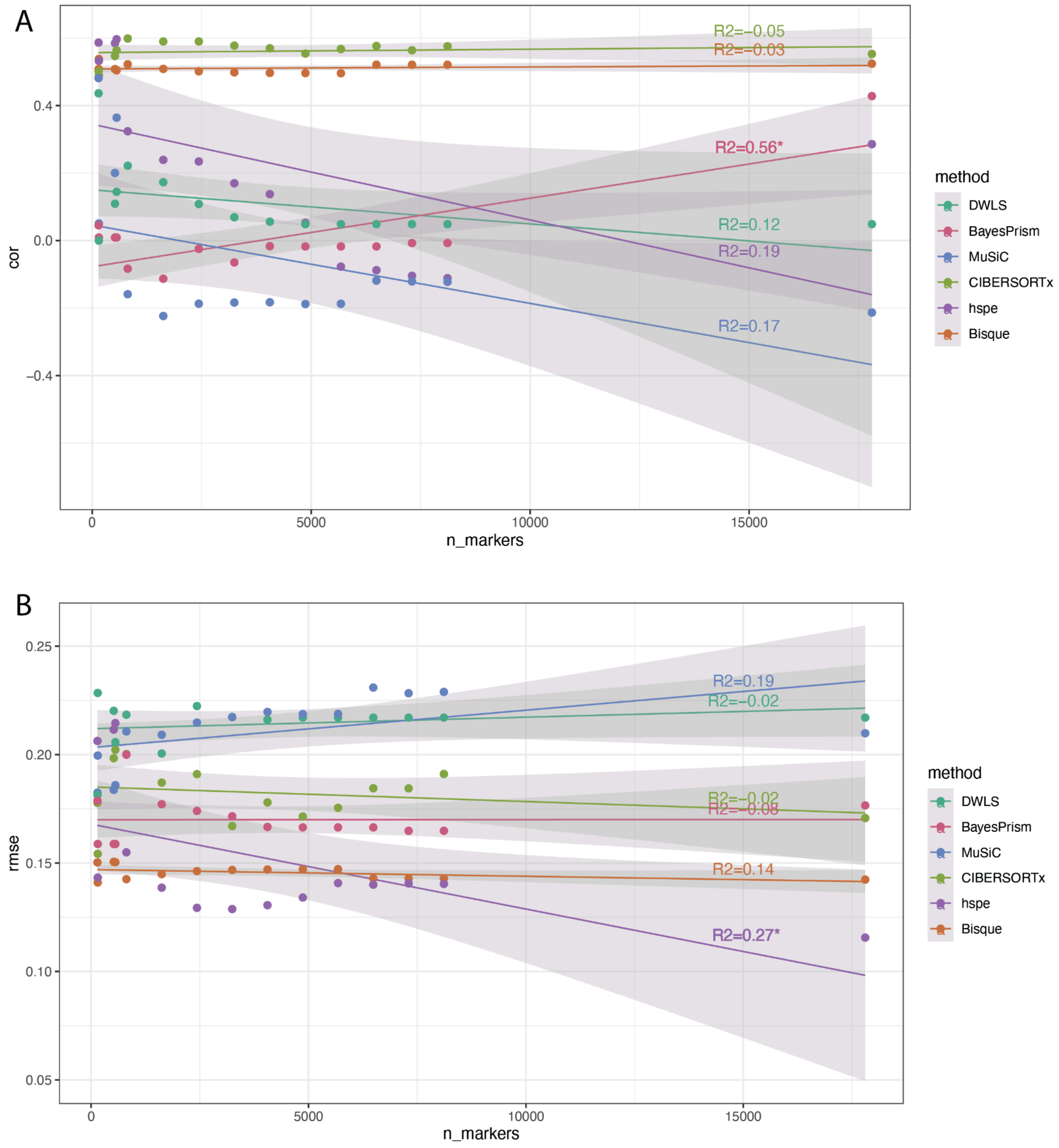

Fig. S27. **Linear fit of number of marker genes and quality metric values for cell type proportion predictions.** Scatter plot of number genes on the x-axis compared to **A.** Pearson's correlation (cor) and **B.** Root mean squared error (rmse) on y-axis for each deconvolution method (color). Adjusted R2 values are added as annotations, linear fits with p-value <0.05 are indicated with "*" (*BayesPrism* for cor, and *hspe* for rmse).

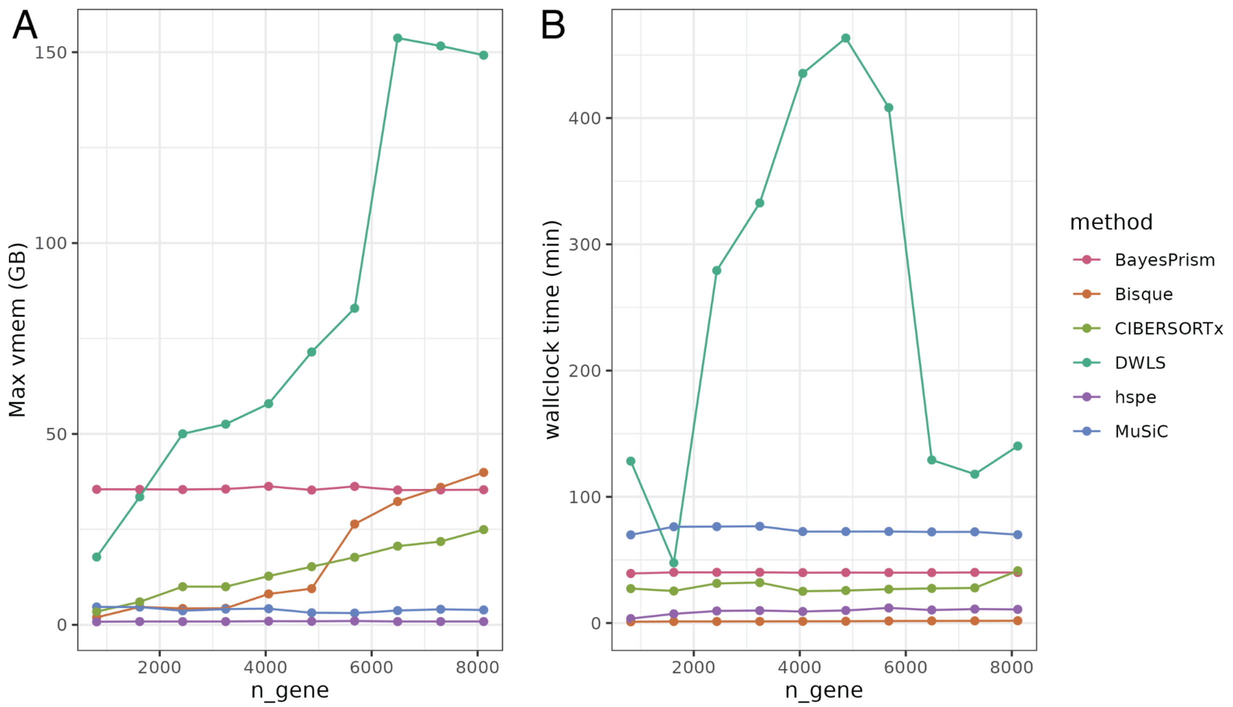

Fig. S28. Method computational efficiency across gene sets. Line plots of A. Maximum virtual memory (max vmem) in gigabytes (GB) and B. wall clock time in minutes used to run the six deconvolution methods (color) over different sets of Highly Variable Genes with the DLPFC snRNA-seq and bulk datasets. DWLS and BayesPrism required significantly more memory (32G or more) and runtime, with DWLS being the slowest and most memory intensive method.

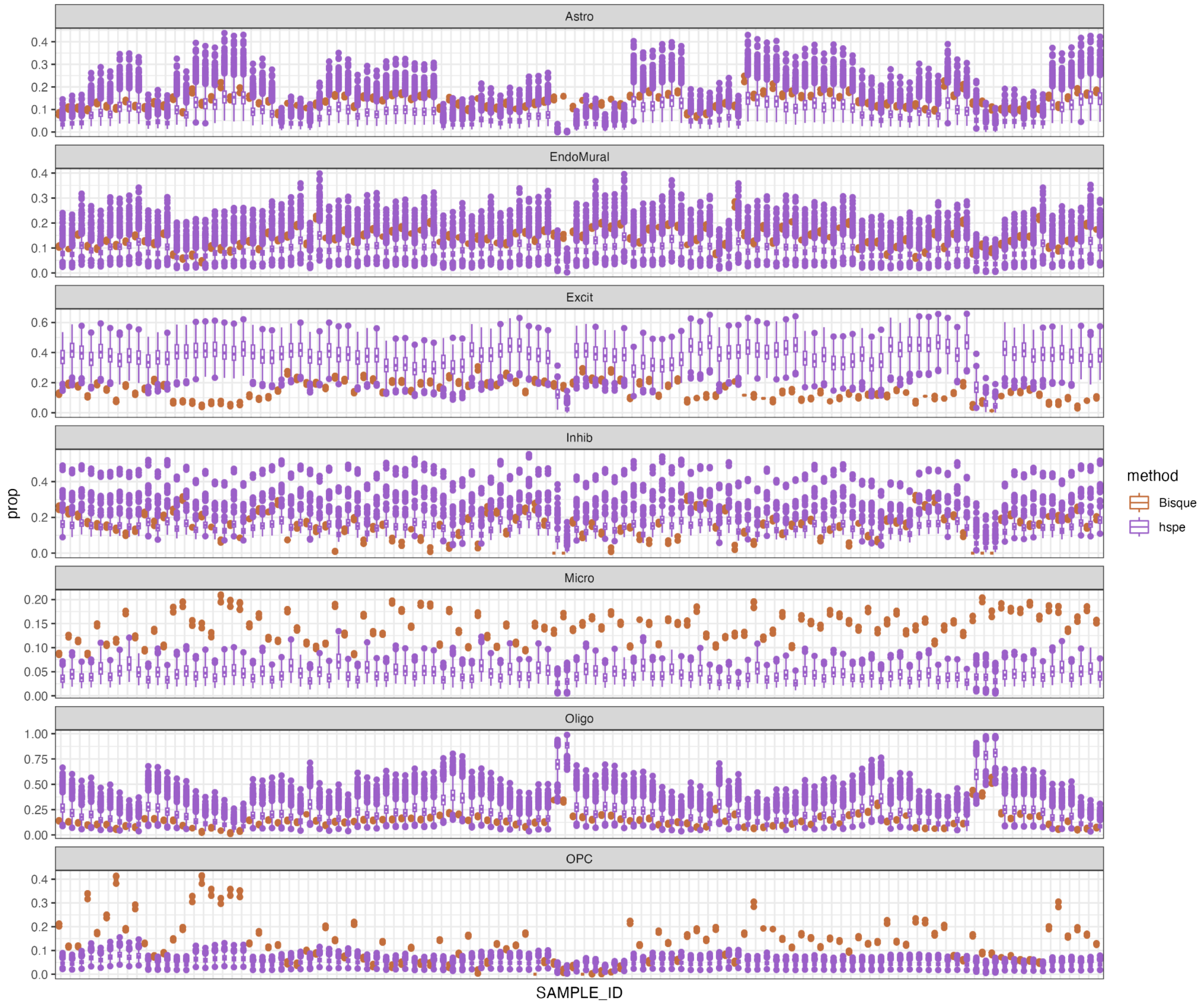

Fig. S29: Simulation results from downsampling to equal input cell proportions for *Bisque* and *hspe*. Boxplots of estimated cell type proportions from simulated equal proportion subsets snRNA-seq reference data, repeated 1,000 times. Deconvolution results shown for all 110 bulk RNA-seq samples (X-axis).

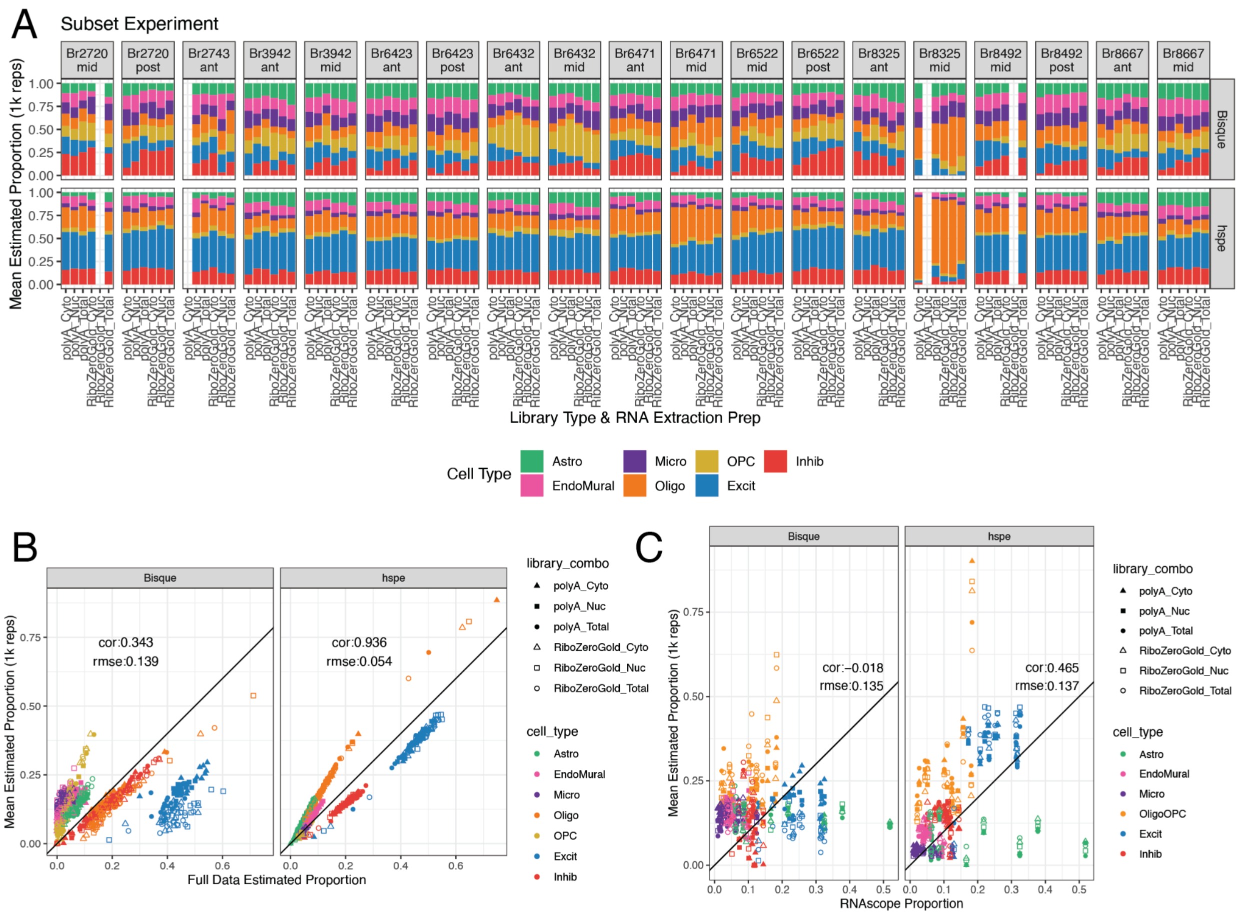

Fig. S30: Performance of *Bisque* and *hspe* on downsampled equal proportion reference data. A. Composition bar plots displaying the mean estimated proportions from the 1,000 sampling replicates for each bulk RNA-seq sample for both methods tested. **B.** Scatter plot of cell type proportions estimated by *Bisque* and *hspe* with full input data vs. the mean predicted cell type proportions by the deconvolution methods under the 1,000 sampling replicates. Points are colored by the cell type and shaped by the combination of bulk RNA-seq RNA extraction method and library type. The annotation lists the overall Pearson's correlation (cor) and root mean squared error (rmse). **C.** Scatter plot of cell type proportions estimated by RNAScope/IF (X-axis) vs. the mean predicted cell type proportions by the deconvolution methods, similar to *B*. Related to **Additional file 1: Fig. S29**.

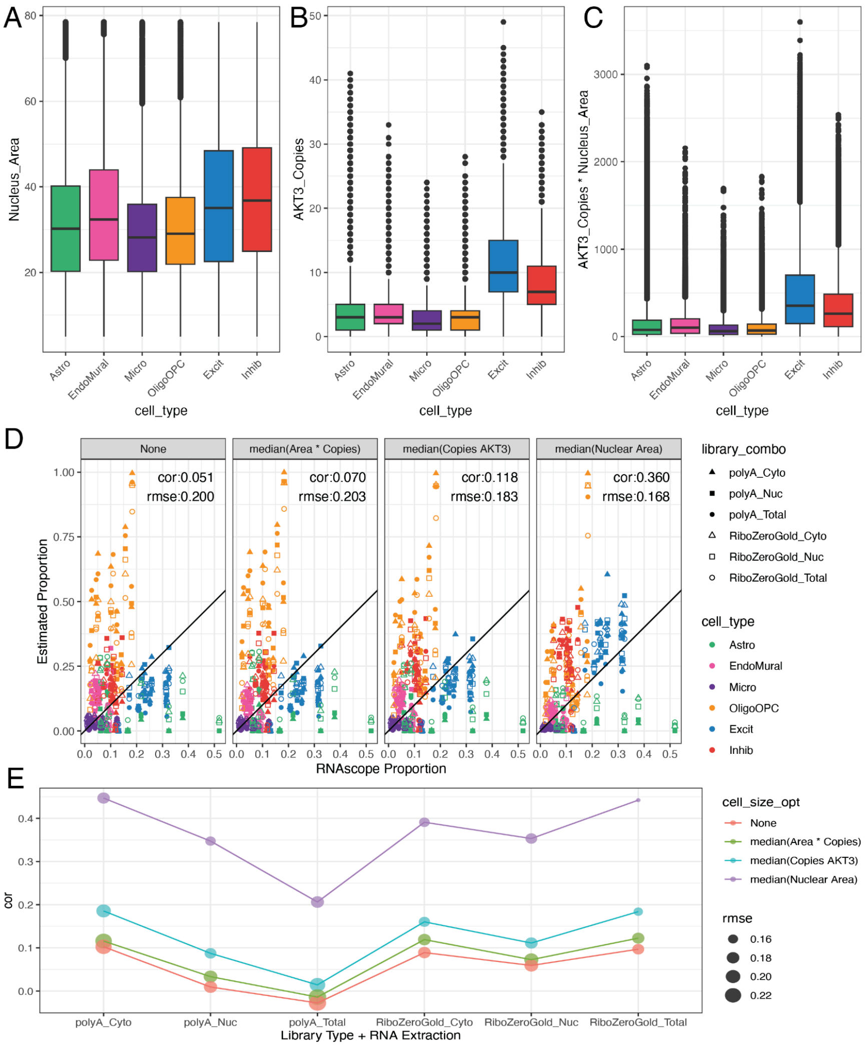

Fig. S31: **Adjusting for cell size with *MuSiC***. Boxplot of the cell size metrics derived from the RNAScope/IF data **A.** nuclear area **B.** Copies of the total RNA expression gene *AKT3*, and **C.** the product of multiplying the nuclear area and number of *AKT3* copies. **D**. Scatter plot of cell type proportions estimated by RNAScope/IF (x-axis) vs. the predicted cell type proportions by *MuSiC* with various cell size metrics. Points are colored by the cell type and shaped by the combination of bulk RNA-seq RNA extraction method and library type. The annotation lists the overall Pearson's correlation (cor) and root mean squared error (rmse). **E**. Correlation (cor) between the predicted proportions by *MuSiC* with cell size metrics and the estimated RNAScope/IF proportions across RNA extraction method and library type combinations, point size reflects the rmse value.

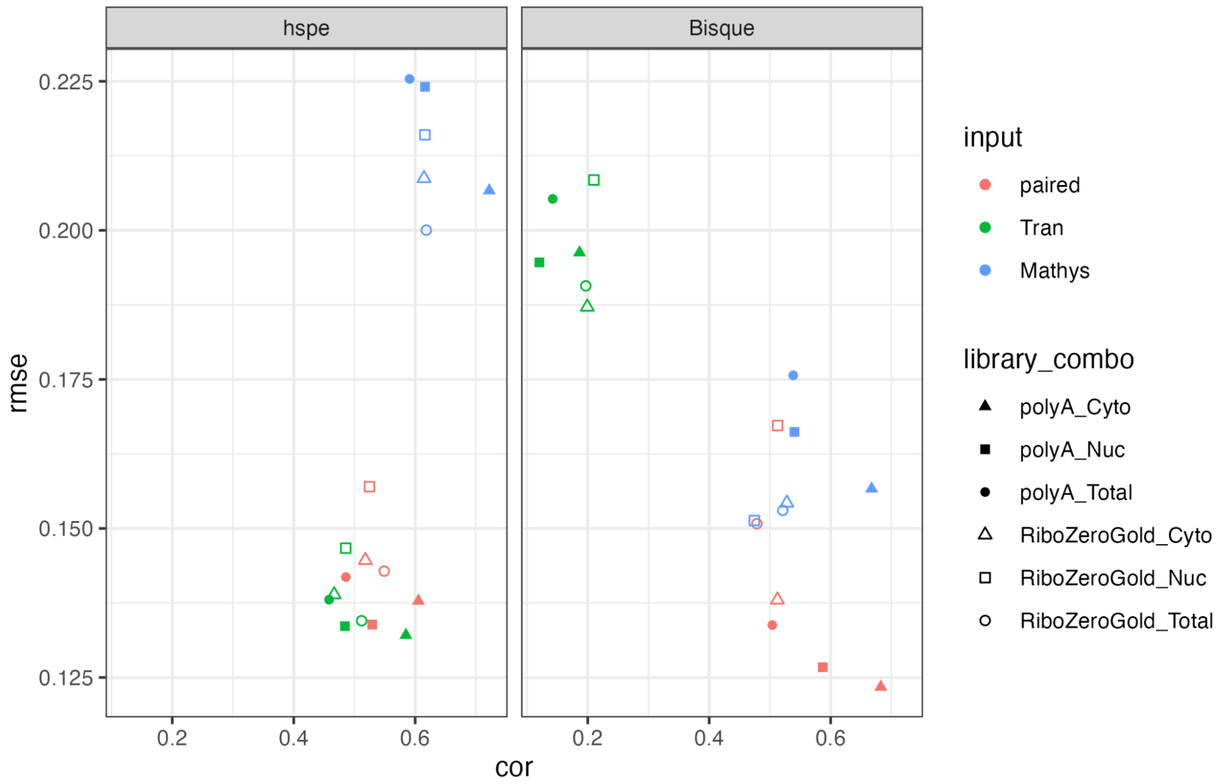

Fig. S32: Scatter plot between the cor and rmse values for cell type proportion predictions across input datasets. Quality metrics for *hspe* and *Bisque* evaluated by bulk RNA-seq RNA extraction method and library type (shape), for the tree tested snRNA-seq input datasets (point color). Related to **Figure 6D-E**.

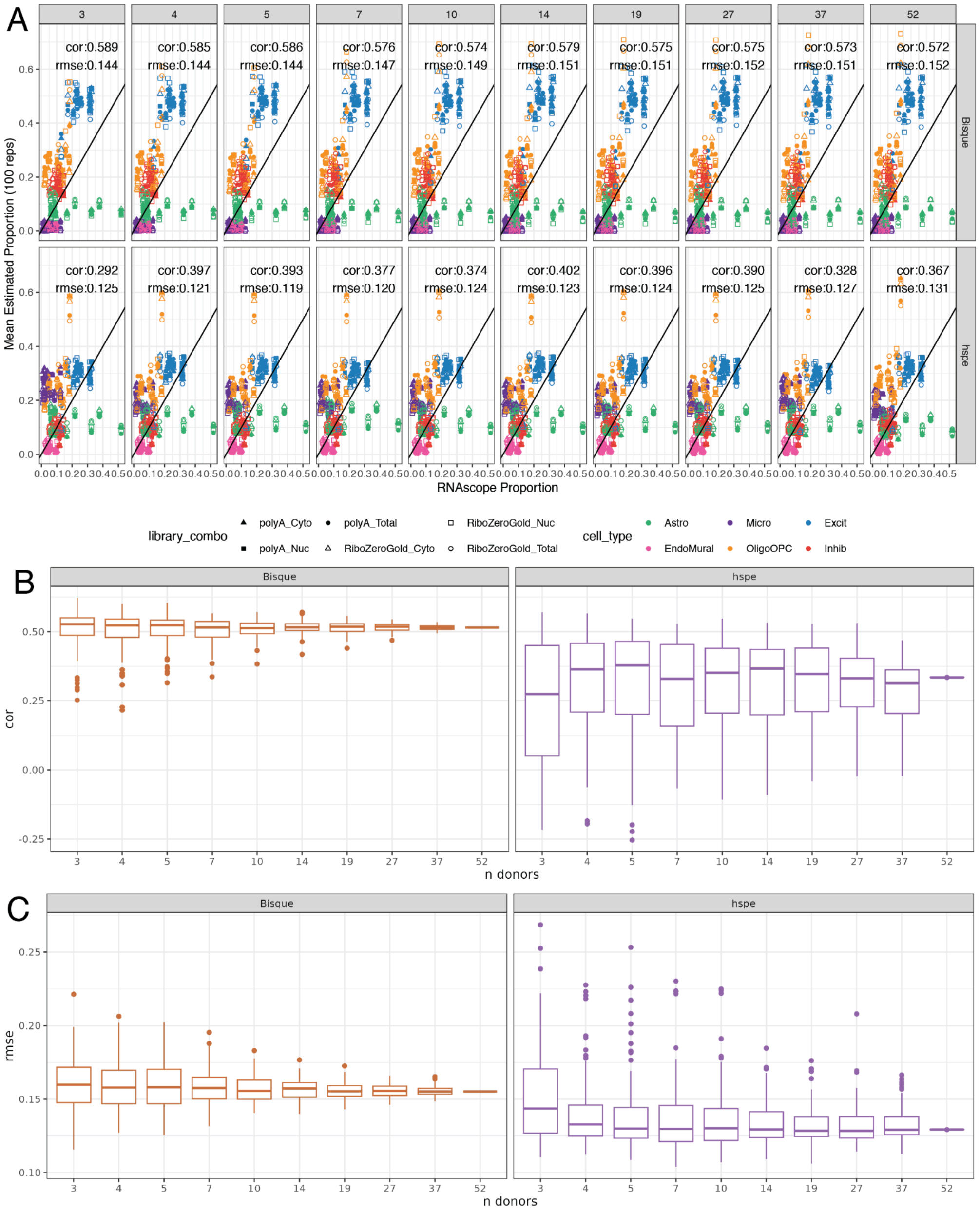

Fig. S33: Performance of *Bisque* and *hspe* on subsets of donors from the CMC DLPFC snRNA-seq dataset. A. Scatter plot of cell type proportions estimated by RNAScope/IF (x-axis) vs. the mean predicted cell type proportions by the deconvolution methods over 100 replicates at different subsets of the total donors. Proportions were calculated with the Mean Ratio top25 marker set. Points are colored by cell type and shaped by the combination of the bulk RNA-seq sample's library type and RNA extraction. Pearson correlation (cor) and root mean squared error (rsme) values are shown for each panel. Boxplots of **B.** Pearson correlation (cor) and **C.** RMSE of predicted proportions vs. RNAScope/IF proportions for the 100 subsets of an increasing number of donors.
