# Supplementary material for: Benchmark of cellular deconvolution methods using a multi-assay dataset from postmortem human prefrontal cortex
Source: Genome Biol. 2025 Apr 7;26:88. doi: 10.1186/s13059-025-03552-3 (PMC11978107; doi:10.1186/s13059-025-03552-3)
Supplement: Supplementary file 3 — Additional file 3. Review history. [file 13059_2025_3552_MOESM3_ESM.docx]

Review History

**First round of review**

**Reviewer 1**

**Were you able to assess all statistics in the manuscript, including the appropriateness of statistical tests used?**

Yes, and I have assessed the statistics in my report.

**Comments to author:**

Estimation of cell type proportions in bulk RNA-seq data using snRNA-seq reference-based deconvolution methods present many challenges. The authors provide a resource for addressing these challenges by generating a multi-assay dataset from adjacent tissue sections across a set of tissue blocks. Different bulk RNA-seq library types and RNA extraction kits were surveyed. The authors use scRNA-sea data as reference datasets to deconvolute bulk RNA-seq data. They propose a novel method for selecting marker genes for cell type deconvolution benchmarking and focus on two key aspects of deconvolution: reference data selection and marker gene selection. The followings are my comments.

Major comments:
1. The author proposes a new method for selecting marker genes for each cell type. They show that the selection of cell type marker genes can affect the deconvolution results. In the main text, the authors use the top 25 genes per cell type estimated from different strategies. How robust are the performance of deconvolution methods when the numbers of top genes vary?
2. In the context of a multi-assay reference deconvolution study, datasets derived from disparate technologies may exhibit varying degrees of batch effects. It is therefore necessary to ascertain whether the observed differences in deconvolution outcomes can be attributed to variations in the reference datasets or to the inherent batch effects.
3. The authors deconvolute the DLPFC tissue with seven major cell types based on the proposed six cell type deconvolution methods. The human DLPFC tissue is a heterogeneous tissue comprising many cell types. Is it possible to look at the more refined cell types (the tissue may consist of more than 30 refined cell types)?
4. The authors employ the multi-assay reference dataset to deconvolute bulk RNA-seq data (related to the title). The results presented in their work are based on the snRNA-seq data (which seems to me just one assay), which serve as the reference data to deconvolute 110 bulk samples, derived from 10 donors. It would be great if the authors could clarify on the meaning of "multi-assay reference dataset".
5. In Figure 4A, the performance of some methods is very poor with close to 0 correlation: "including MuSiC (cor=0.051,rmse=0.2), BayesPrism (cor=0.009, rmse=0.159), and DWLS (cor=0, rmse=0.228)". It will be helpful to provide more discussion to provide some insight on why these methods perform so poor.

Minor comments：
6. It seems that Table S1 just contain the links for the data. It would be great to summarize some key information of the 10 donors.
7. It would be great if the authors check thoroughly that the references are correct and appropriate. For example, in the section "multi-modal dataset from human DLPFC", authors mention that "Some deconvolution algorithms take into account the different statistical properties of measuring gene expression with bulk RNA-seq and snRNA-seq assay". It would be better to be more specific with clear reference.
8. In Figure S6, there is a typo. It should be 'Each' instead of 'each'.

**Reviewer 2**

**Were you able to assess all statistics in the manuscript, including the appropriateness of statistical tests used?**

Yes, and I have assessed the statistics in my report.

**Comments to author:**

The manuscript, titled "Benchmarking Cellular Deconvolution Methods Using a Multi-Assay Reference Dataset from Postmortem Human Prefrontal Cortex," addresses the challenge of estimating cell type composition in heterogeneous tissues, particularly the human brain, through cellular deconvolution of bulk RNA-sequencing (RNA-seq) data with single-cell or nuclei RNA-seq (sc/snRNA-seq) references. The study provides a comprehensive multi-assay dataset from postmortem human dorsolateral prefrontal cortex (DLPFC), encompassing spatially-resolved transcriptomics, snRNA-seq, bulk RNA-seq, and RNAScope/Immunofluorescence (RNAScope/IF). It introduces a Mean Ratio method for selecting cell type marker genes and evaluates six different deconvolution algorithms, with Bisque and hspe emerging as the most accurate and robust methods. However, challenges such as cell size differences, marker gene quantification variability, and reference snRNA-seq cell composition influence the accuracy of deconvolution. Despite the relevance of the topic and the approach, there are several critical issues that require resolution before considering the paper for publication.

Overall Assessment:

This paper explores various deconvolution methods, yet it does not introduce a novel methodology; rather, it adopts the Mean Ratio method to select deconvolution marker genes and evaluate existing methods. The dataset generated in this paper is useful for the purposed outlined in the paper. The R package called DeconvoBuddies introduced in this paper provides helper functions for the deconvolution process. While it is quite useful for bioinformaticians for choosing the appropriate deconvolution method for themselves, there are some limitations in the inherent methodology used in this article. Below, I have provided detailed feedback on the major concerns.

Major Issues:

1. Deconvolution methods for scRNA-seq are not new and there are a multitude of methods already available to perform this task. Many of them already perform relatively well. There are already many baseline comparisons of these tools too. Providing additional tools for this task and/or baseline comparisons is not novel despite a very useful evaluation dataset.
2. In PDF page 11, The Mean Ratio method is presented as the novelty in this article. However, in assessing the Mean Ratio method for identifying marker genes with robust cell type-specific expression, several issues emerge. Firstly, this method considers the mean expression of genes which may not fully capture the intricacies of gene expression patterns within diverse cell types. This could potentially overlook nuances such as bimodal or multimodal expression distributions, leading to an oversimplified representation of cell type specificity. Also, it doesn't consider the variability of the genes.

3. Under Section "RNAScope/Immunofluorescence Data Generation and HALO Analysis", in PDF page 28, Line 49: "Segmentation was optimized for accurate identification of punctate AKT3 RNAScope/IF signals as well as each cellular object for excitatory neurons (SLC17A7), inhibitory neurons (GAD1), astrocytes (GFAP), microglia (TMEM119), a combined class of oligodendrocytes and oligodendrocyte precursor cells [OligoOPC] (OLIG2), and endothelial/mural cells (Claudin 5: CLDN5), using user-defined size and intensity thresholds." The potential issue with segmentation optimization using user-defined size and intensity thresholds is the risk of introducing bias and overlooking biological variability, which may lead to inaccuracies in cell type identification and subsequent analysis. This is a need for proper explanation on justifying the threshold values.

4. Similarly, in the same section, in PDF page 29, Line 9, "The same thresholds were applied to a given cell type across all tissue samples, regardless of the donors, including the size thresholds for nucleus and cytoplasm." The use of same thresholds for nucleus and cytoplasm across all tissue samples, regardless of donor variations, might not account for biological differences between samples. This could lead to inaccurate cell type quantifications.

5. In the Section "Evaluation of Deconvolution Results" PDF page 32, line 39, the use of the Pearson correlation as evaluation of the method has several limitations, such as it assumed linear relationship, normality etc. Other methods of evaluation such as Concordance between the groups, or Spearman's rank correlation coefficient would have been more appropriate. These alternatives can offer a more nuanced and robust evaluation of deconvolution results, leading to more reliable conclusions.


Conclusion:
In summary, while the paper addresses a relevant topic and presents a quality dataset of the postmortem human prefrontal cortex, it does not provide a novel deconvolution approach. The Mean Ratio method has some fundamental issues which are mentioned in the detailed comments above. Significant revisions are necessary to improve its methodological rigor and clarity.

**Reviewer 3**

**Were you able to assess all statistics in the manuscript, including the appropriateness of statistical tests used?**

Yes, and I have assessed the statistics in my report.

**Comments to author:**

This manuscript presents a comprehensive benchmark of six bulk RNA-seq deconvolution methods using a novel dataset specifically generated for this benchmark. The dataset comprises snRNA-seq, RNAScope/IF, Visium, and bulk RNA-seq from 22 tissue blocks, utilizing different RNA extraction protocols. Previous benchmark studies have been inconclusive, likely due to variations in study design, including differences in the characteristics of reference scRNA-seq datasets, marker gene selection, and simulation approaches. While this benchmark may not address all questions, it provides valuable insights and the authors are to be commended for their diligent work. Independent benchmarks are often particularly valuable for the field, as are reference datasets with a carefully derived "gold standard".

I have a few comments that will hopefully help to further improve the manuscript. The first comment addresses what I believe is an important question that has not been fully covered in the benchmark study. The additional comments are either for clarification or pertain to less important issues.

1) Towards the end of the manuscript, the authors assess the performance of Bisque and hspe when non-matched external snRNA-seq reference data from the same brain region (DLPFC) is used. They speculate that the smaller number of nuclei in the Tran et al. reference may contribute to Bisque's poorer performance with this reference, but also note the different cell type composition of this dataset. I believe this is a highly relevant question that warrants further investigation: How large should an snRNA-seq reference dataset be to achieve good deconvolution results? There are much larger DLPFC reference datasets with over 1.5 million nuclei (Kellis/Tsai Cell paper and the De Jager Nature Genetics paper) that could be used to evaluate the methods' performances with increasing reference dataset size. These datasets should be large enough to observe convergence well before the entire dataset is used.

2) Regarding the RNAscope/IF cell type proportions, it is unclear why so many cells are classified as "other." For instance, in Fig 2D sample BR8667_mid has about 50% of cells classified as Ast/Mic/Exc/Inh/Oli/End and the other half as "other cells." Does this reflect high uncertainty in the RNAscope/IF-based cell type classification? This should be explained in more detail. Considering these results, the authors should also discuss why they prefer RNAscope/IF as the "gold standard" over snRNA-seq. It is not obvious to me which of these two methods yields more accurate cell type proportion estimations, as both have potential biases. Perhaps it would be more appropriate to consider both references equally.

3) Why do all methods demonstrate poor estimations for astrocytes? At the same time, the RNAscope-derived astrocyte proportions seem overestimated for some samples. Can the authors speculate on why the methods don't work well for astrocytes, or whether this may be caused by the reference?

4) The benchmark demonstrates the sensitivity of some methods to the selection of marker genes. I agree with the approach of comparing all methods using the same set of marker genes, as performed in the present study. However, it would also be beneficial to run each method once using the exact marker gene selection procedure suggested by the authors of the respective methods (as listed in Table 1) and report those results either in Table 1 or in the supplement.

5) Can the authors clarify whether section BR8325_mid has a very large fraction of WM, or if it is an outlier that results in an overestimation of oligodendrocytes by all methods?

6) The laminar structure of the cortex can vary considerably. The sections used in this study to generate the bulk RNA-seq, RNAscope, and snRNA-seq data were sandwiched between two Visium sections (Fig 1A). It is fairly easy to detect WM/GM using Visium ST data. Have the authors considered estimating the proportion of WM at both ends of the tissue block using their existing Visium data to determine how much of the cell type proportion errors may be attributable to changes in the laminar structure along the tissue block?

7) Finally, I would like to encourage the authors to prepare a Bioconductor data package including the processed bulk RNA-seq and snRNA-seq data, as well as the results (cell type proportions) from the RNAscope experiments. Such a package would further facilitate the use of this benchmark dataset in future studies.

**Authors Response**

**Point-by-point responses to the reviewers’ comments:**

Please find below our detailed response to Reviewers. Reviewer comments are in black and responses are in blue. In the revised manuscript, we have highlighted all new text in red.

Reviewer reports:

# Reviewer: 1

**Estimation of cell type proportions in bulk RNA-seq data using snRNA-seq reference-based deconvolution methods present many challenges. The authors provide a resource for addressing these challenges by generating a multi-assay dataset from adjacent tissue sections across a set of tissue blocks. Different bulk RNA-seq library types and RNA extraction kits were surveyed. The authors use scRNA-sea data as reference datasets to deconvolute bulk RNA-seq data. They propose a novel method for selecting marker genes for cell type deconvolution benchmarking and focus on two key aspects of deconvolution: reference data selection and marker gene selection. The followings are my comments.**

We would like to thank the reviewer for their time reading our manuscript and providing comments. Accordingly, we have updated the manuscript to help clarify these points to our future readers. Thank you for your constructive input.

**Major comments:**

**1. The author proposes a new method for selecting marker genes for each cell type. They show that the selection of cell type marker genes can affect the deconvolution results. In the main text, the authors use the top 25 genes per cell type estimated from different strategies. How robust are the performance of deconvolution methods when the numbers of top genes vary?**

We evaluated the performance of the six methods on five different sets of genes (1vALL top25 n=145, Mean Ratio top25 n=151, Mean Ratio MAD3 n=520, Mean Ratio over2 n=557, and the FULL set of common genes n=17804. The performance of the methods with different gene sets is shown in **Figure 5** and **Fig S23**. However these figures don’t directly compare accuracy vs. the number of genes used. To address this point, we’ve included a new supplementary figure (**Fig S26**), which plots the correlation of each method against the number of genes categorically (**Fig S26A**) and continuously (**Fig S26B**).

In **Fig S26**, we again observe that method performance can vary widely with different sets of marker genes: *hspe* has much lower correlation with the FULL set of marker genes, *CIBERSORTx* and *Bisque* are consistently top performers across marker sets, *BayesPrism* improves with FULL set vs. selected makers, and MuSiC shows inconsistent performance. Testing for a linear association between the number of genes and deconvolution performance (cor) through a simple regression applied to these five data points, only *hspe* and BayesPrism have models with statistically significant fits (p-value < 0.05), and even then the predicted ß are extremely small. As accuracy in performance seems to be based on the deconvolution method and gene selection method rather than the number of genes, we do not think that further exploring incremental sets of marker genes will yield more clarity in pipeline selection for researchers.

We have added this sentence to the results section *Benchmark of Deconvolution Methods with Different Gene Sets*:

For most methods, the relationship between number of marker genes and correlation is not linear (**Fig S26**), and accuracy cannot be simply predicted from the number of genes.

**Fig S26: Scatter plot of number of marker genes and correlation values for cell type proportion predictions.** Pearson correlation (cor) between the predicted proportions by deconvolution methods and the estimated RNAScope/IF proportions for all library type combinations over the five tested marker gene sets, compared to the number of genes in each gene set listed in top annotations. **A.** Plotted with marker gene sets on the x-axis shown categorically arranged by number of genes, and data points connected with lines. **B.** Plotted with marker gene sets on the x-axis plotted by the number of marker genes, the lines show linear fit with a listed R2 value.

**2. In the context of a multi-assay reference deconvolution study, datasets derived from disparate technologies may exhibit varying degrees of batch effects. It is therefore necessary to ascertain whether the observed differences in deconvolution outcomes can be attributed to variations in the reference datasets or to the inherent batch effects.**

We agree that batch effects are important to consider. In the downstream analysis of this dataset, many measures were taken to avoid batch effects. For example, the snRNA-seq data was batch corrected using Harmony, see (Fig S23 from Huuki-Myers et al.). We have expanded the methods to explain more details about sequencing batches. We argue that any batch effects between assays that remain in this dataset, despite the precautions taken in the experimental design, will still be minor compared to the scenario where deconvolution is performed without any paired data sets (which is the more typical situation for users). To address batch effects in the manuscript, we have added details to the following sections:

Added the following Supplementary Figure:

[**Fig S4**](https://docs.google.com/document/d/1Rk5Z8iyekZoVaYxqjl4nIHx9wTo4p8GjR1J1vki-9Ow/edit#sfigu_bulk_PCA_qc)**: Bulk RNA-seq data Principal Component Analysis (PCA). A.** PC1 versus 2 colored by sequencing round **B.** Boxplots of The PC values by library type and sequencing round.

Added related text to the following sections:

Methods: Cryosectioning and Tissue Sample Collection of Orthogonal Datasets:

During data generation, we took precautions to minimize batch effects. All assays were performed by the same experimenter. Furthermore tissue for all assays was cut in the same cryosectioning session. For data collection, donors were divided into 5 sex balanced groups (4 batches of 4, 1 batch of 3, n=19). These batches were used during cryosectioning, snRNAseq, and RNAscope/IF experiments (sections for each batch were mounted on the same slide).

For bulk experiments, these groups were merged and run in 2 batches, which were inherently balanced due to the experimental design described above. It should also be noted that nuclear and cytoplasmic bulk RNA fractions are run on the same column within the same extraction.

Results: Multi-modal Dataset from Human DLPFC

The sequencing round did not drive large changes in the gene expression, observed in the top 6 PCs (**Fig S4**).

**3. The authors deconvolute the DLPFC tissue with seven major cell types based on the proposed six cell type deconvolution methods. The human DLPFC tissue is a heterogeneous tissue comprising many cell types. Is it possible to look at the more refined cell types (the tissue may consist of more than 30 refined cell types)?**

The reviewer is correct that the cell types in the DLPFC can be divided beyond the seven broad cell types we utilized in this study. The snRNA-seq data in this study was originally found to have 29 distinct cell type clusters (Huuki-Myers et al., 2024). However our orthogonal RNAScope/IF dataset was limited to measuring only six cell types, therefore we restricted our analysis to broad cell types to best account for the major cell populations in the DLPFC.

This resolution is similar to cell type resolutions evaluated in prior deconvolution benchmark studies in the brain and other tissue types: Dai et al., 2024 uses 7-8 cell types in their reference snRNA-seq datasets and 5 cell types in the Immunohistochemistry data set (Dai et al. Table 1.), and Cobos et al., 2020 considers five reference snRNA-seq considering 5-10 cell types.

We have added a sentence to the discussion adding this comparison to the literature:

“This cell type resolution is similar to those evaluated in prior deconvolution benchmarks (5-10 cell types) [(Avila Cobos et al. 2020; Dai et al. 2023)](https://sciwheel.com/work/citation?ids=10008140,14539797&pre=&pre=&suf=&suf=&sa=0,0).”

As observed in the Cobos et al. 2020 benchmark, solving for more and rarer cell types is a more challenging problem with decreasing accuracy. As only *Bisque* and *hspe* could reliably (but still imperfectly) predict cell type compositions at the broad cell type resolution,we feel evaluating a much finer cell type resolution would likely not better inform pipeline selection for readers.

**4. The authors employ the multi-assay reference dataset to deconvolute bulk RNA-seq data (related to the title). The results presented in their work are based on the snRNA-seq data (which seems to me just one assay), which serve as the reference data to deconvolute 110 bulk samples, derived from 10 donors. It would be great if the authors could clarify on the meaning of "multi-assay reference dataset".**

The phrase "multi-assay reference dataset" only appears in the title of the paper. The use of “reference” in the title was meant to describe that the full dataset could be used as a resource to evaluate deconvolution methods. As the reviewer correctly points out, the word “reference” could also be interpreted as the “reference” snRNA-seq dataset. We do not provide multiple reference snRNA-seq datasets, so we understand how this is confusing. To improve the understanding of the title, we have removed the word “reference” and the updated title reads:

**Benchmark of cellular deconvolution methods using a multi-assay dataset from postmortem human prefrontal cortex**

**5. In Figure 4A, the performance of some methods is very poor with close to 0 correlation: "including MuSiC (cor=0.051,rmse=0.2), BayesPrism (cor=0.009, rmse=0.159), and DWLS (cor=0, rmse=0.228)". It will be helpful to provide more discussion to provide some insight on why these methods perform so poor.**

The Mean Ratio top 25 cell type proportion predictions from *DWLS*, *MuSiC*, and *BayesPrism* are all in disagreement with RNAScope/IF (best *MuSiC*, cor = 0.052) and snRNA-seq (best *MuSiC*, cor = 0.294) proportion predictions, but are similar to each other’s estimates. The correlation between the three methods estimates is relatively high: *DWLS* vs. *MuSiC*=0.886, *DWLS* vs. *BayesPrism*=0.601, and *MuSiC* vs. *BayesPrism* = 0.735 (see **Fig S17** for more details). As *DWLS* and *MuSiC* both use weighted least squares their agreement makes sense.

Subsetting to specific cell types, compared to RNAScope/IF the methods also follow similar patterns. They are weak at predicting OligoOPC and Excit (cor > 0.2), but fail (cor < 0.2) on the other cell types, specifically Astro and EndoMural. OligoOPC and EndoMural cell types are often over-predicted, and Excit and Astro are often under-predicted by these methods.

We have added to the discussion the following sentence:

While *BayesPrism*, *MuSiC*, and *DWLS* results had low correlation against RNAScope/IF and snRNA-seq, they have higher correlation among themselves, particularly among Excit and OligoOPC (**Fig S17**). *MuSiC* and *DWLS* are both using weighted least squares, which could explain the similarity in their results (**Fig S17**).

**Minor comments：**

**6. It seems that Table S1 just contain the links for the data. It would be great to summarize some key information of the 10 donors.**

Thank you for the suggestion, we agree it would be helpful to add. We have added a new Supplementary Table (**Table S1**) with the age, sex, and primary diagnosis of the ten donors in this study. The number of samples for each assay, pre and post QC are listed as well.

**7. It would be great if the authors check thoroughly that the references are correct and appropriate. For example, in the section "multi-modal dataset from human DLPFC", authors mention that "Some deconvolution algorithms take into account the different statistical properties of measuring gene expression with bulk RNA-seq and snRNA-seq assay". It would be better to be more specific with clear reference.**

We have edited this sentence to reference *Bisque* specifically.

“Some deconvolution algorithms, such as *Bisque* [(Jew et al. 2020)](https://sciwheel.com/work/citation?ids=8798196&pre=&suf=&sa=0), take into account the different statistical properties of measuring gene expression with bulk RNA-seq and snRNA-seq assays.”

**8. In Figure S6, there is a typo. It should be 'Each' instead of 'each'.**

Thank you for noticing this typo. We have fixed it in the new version.

# Reviewer: 2

**Review**

**The manuscript, titled "Benchmarking Cellular Deconvolution Methods Using a Multi-Assay Reference Dataset from Postmortem Human Prefrontal Cortex," addresses the challenge of estimating cell type composition in heterogeneous tissues, particularly the human brain, through cellular deconvolution of bulk RNA-sequencing (RNA-seq) data with single-cell or nuclei RNA-seq (sc/snRNA-seq) references. The study provides a comprehensive multi-assay dataset from postmortem human dorsolateral prefrontal cortex (DLPFC), encompassing spatially-resolved transcriptomics, snRNA-seq, bulk RNA-seq, and RNAScope/Immunofluorescence (RNAScope/IF). It introduces a Mean Ratio method for selecting cell type marker genes and evaluates six different deconvolution algorithms, with Bisque and hspe emerging as the most accurate and robust methods. However, challenges such as cell size differences, marker gene quantification variability, and reference snRNA-seq cell composition influence the accuracy of deconvolution. Despite the relevance of the topic and the approach, there are several critical issues that require resolution before considering the paper for publication.**

**Overall Assessment:**

**This paper explores various deconvolution methods, yet it does not introduce a novel methodology; rather, it adopts the Mean Ratio method to select deconvolution marker genes and evaluate existing methods. The dataset generated in this paper is useful for the purposed outlined in the paper. The R package called DeconvoBuddies introduced in this paper provides helper functions for the deconvolution process. While it is quite useful for bioinformaticians for choosing the appropriate deconvolution method for themselves, there are some limitations in the inherent methodology used in this article. Below, I have provided detailed feedback on the major concerns.**

We would like to thank the reviewer for their time reading our manuscript and providing comments. Accordingly, we have improved the manuscript to help clarify these points to our future readers. While we are not presenting a novel deconvolution method, we do consider our study design, data, and results to be a novel approach to benchmarking deconvolution methods.

**Major Issues:**

**1. Deconvolution methods for scRNA-seq are not new and there are a multitude of methods already available to perform this task. Many of them already perform relatively well. There are already many baseline comparisons of these tools too. Providing additional tools for this task and/or baseline comparisons is not novel despite a very useful evaluation dataset.**

We agree with the reviewer that there has been much written on deconvolution and many attempts to identify the most accurate methods. However we have found the literature and existing benchmarks to be inconclusive, often presenting conflicting rankings of method performance. We note these mixed conclusions in Table 1 in the “Top Benchmark Performance” column, and in the background section.

 Benchmarking results from different papers on non-simulated data:

- *MuSiC* paper (Wang et al., *Nat. Commun.*, 2019): *MuSiC* > *NNLS* > *BSEQ-sx* > *CIBERSORT*
  - Pancreatic Islet: Beta cells vs. HbA1c (Fig 2a)
- *Bisque* paper (Jew et al., *Nat. Commun.*, 2020): *Bisque* > *MuSiC* > *CIBERSORT*
  - DLPFC: Microglia vs. Braak stage, Neuron vs. Cognitive diagnostic category (Fig 4)
- Cobos et al., *Nat. Commun.*, 2020 benchmark: *DWLS* > *MuSiC* > *Bisque* > *deconvoSeq*
  - Human PMBC flow sorted (Fig 7)
- Jin et al., *Genome Biol.*, 2021 benchmark: *CIBERSORT*, *MuSiC* > *EPIC*, *TIMER*, *DeconRNAseq*
  - Human Whole Blood, simulations
- Dai et al., *Science*, 2024 benchmark: *Dtangle (hspe)* > *Bisque* > Other Methods
  - human brain IHC & scRNA-seq data

Also few of the benchmark experiments were tested on brain data, and as noted by Cobos et al. 2020, different methods perform best on different data sets. This motivated us to explore the performance of the top deconvolution methods specifically in brain tissue, which presents unique challenges given its diversity of cell types. We believe that this study provides a novel and rich dataset for such an evaluation. Using a different approach, we also draw a similar conclusion as the recent Dai et al. 2024 benchmark, thereby building a consensus on the best performing deconvolution methods for postmortem human brain research.

**2. In PDF page 11, The Mean Ratio method is presented as the novelty in this article. However, in assessing the Mean Ratio method for identifying marker genes with robust cell type-specific expression, several issues emerge. Firstly, this method considers the mean expression of genes which may not fully capture the intricacies of gene expression patterns within diverse cell types. This could potentially overlook nuances such as bimodal or multimodal expression distributions, leading to an oversimplified representation of cell type specificity. Also, it doesn't consider the variability of the genes.**

Marker gene selection is a challenging problem, as genes have complex patterns of expression across cell types. We agree that the MeanRatio method does not account for multimodal expression, but this is also a common limitation in marker finding on methods relying on *t*-tests or Wilcoxon tests, among other ones.

To detect unexpected patterns in gene expression as the reviewer pointed out, we recommend performing exploratory data analysis of the putative marker genes. In more detail, we recommend making violin gene expression plots for these genes. The violin plots for this data are available in our code repository at <https://github.com/LieberInstitute/Human_DLPFC_Deconvolution/blob/main/plots/06_marker_genes/04_marker_gene_plots/marker_expression_mean_ratio_broad.pdf>. We did not observe any complex gene expression patterns that would confound the MeanRatio marker finding in the top marker genes for this DLPFC dataset (**Fig 3D**). The other marker genes can be observed through the interactive *iSEE* website <https://libd.shinyapps.io/spatialDLPFC_snRNA-seq/> listed at <https://research.libd.org/spatialDLPFC/#interactive-websites>.

The 1vALL method, which is ultimately based on *t*-tests, does consider variance where MeanRatio does not. However we've observed that the Mean Ratio method consistently selects a subset of genes with high standardized log fold changes (std.logFC) (that is, *t*-statistics as discussed at <https://github.com/MarioniLab/scran/issues/57>) from 1vALL (**Fig 3C**), and selects more cell type specific marker genes (**Fig 3E-F**). With larger samples, such as a reference datasets with a large number of nuclei and broad cell types, the mean expression of a cell type becomes a stable estimator as by the Law of Large Numbers under a no-heavy tails assumption. However in cases where there are very few cells per cell type, the stability of the mean estimator decreases. Following the recommendation at <https://bioconductor.org/books/3.19/OSCA.multisample/multi-sample-comparisons.html#performing-the-de-analysis> which cites <http://dx.doi.org/10.1038/s41467-020-19894-4> we have implemented a warning to DeconvoBuddies::get_mean_ratio()that checks that at least 10 cells per cell type are present. This is a strictly minimum requirement, though a higher number of cells per cell type is much preferable. We also recommend users to compare the 1vALL logFC against MeanRatio (as we did in **Fig 3C**), as low genes with a low logFC but high MeanRatio could be worth inspecting more closely.

In summary, we have documented this step in the marker finding tutorial and provided plotting functions for plotting gene expression in the DeconvoBuddies package. See <https://research.libd.org/DeconvoBuddies/articles/Marker_Finding.html> for more details. We encourage users to observe the expression patterns of markers, potentially exclude problematic genes, and provide a warning to users of potential instability in the Mean Ratio results when they have very few cells per cell type. See the cellType_col parameter information at <https://research.libd.org/DeconvoBuddies/reference/get_mean_ratio.html> and <https://github.com/LieberInstitute/DeconvoBuddies/blob/4192530e91e294ddfd25e1e6b09e1c2a890dd574/R/get_mean_ratio.R#L86>.

**3. Under Section "RNAScope/Immunofluorescence Data Generation and HALO Analysis", in PDF page 28, Line 49: "Segmentation was optimized for accurate identification of punctate AKT3 RNAScope/IF signals as well as each cellular object for excitatory neurons (SLC17A7), inhibitory neurons (GAD1), astrocytes (GFAP), microglia (TMEM119), a combined class of oligodendrocytes and oligodendrocyte precursor cells [OligoOPC] (OLIG2), and endothelial/mural cells (Claudin 5: CLDN5), using user-defined size and intensity thresholds." The potential issue with segmentation optimization using user-defined size and intensity thresholds is the risk of introducing bias and overlooking biological variability, which may lead to inaccuracies in cell type identification and subsequent analysis. This is a need for proper explanation on justifying the threshold values.**

We agree with the reviewer that image segmentation optimization is a challenging problem that is prone to experimenter bias. Here we used HALO (Indica labs) FISH/IF module for quantification, which requires that each tissue section be segmented and quantified independently. For consistency, we used the same approach for segmentation of each tissue section. We clarify in the manuscript the thresholds that HALO requires a user set as detailed below at *Methods: RNAScope/Immunofluorescence Data Generation and HALO Analysis*:

Based on our previous experience with nuclear segmentation in DLPFC (Maynard et al, *Nucleic Acids Research*, 2020), nuclear size was set in a range from 5um² to 200um² to avoid inclusion of nuclear fragments and debris. Cytoplasmic size was set a dilation of 4.2um around the segmented nucleus to include some cytoplasmic transcripts with high confidence of belonging to that cell (i.e. to include an axon hillock for neurons). We aimed to be conservative so as not to capture transcripts from adjacent cells or passing fibers. Nuclear segmentation parameters were defined to capture single cells and split cell aggregates. Beyond these user-defined parameters, which are required to run HALO, phenotype algorithms were optimized based on staining intensity to avoid overcalling and undercalling cell types. Each phenotype algorithm was optimized across several randomly selected areas within multiple donors before finalizing the threshold for that cell type. These phenotypes were utilized in the majority of donors. In some cases, phenotype algorithms had to be adjusted to accommodate variability across donors, such as higher background, intense staining, or changes in cell density. For example, astrocytes were often overcalled in white matter regions, which interfered with the detection of oligodendrocyte, microglia, and endothelial/mural cell signals, leading to false positives. In tissue sections where the default algorithms did not perform optimally, we optimized the algorithms to represent the cell type distribution as accurately as possible.

Finally, we would like to note that our lab has extensive experience in segmentation and cell phenotyping of postmortem DLPFC tissue using different approaches, including machine learning approaches (Maynard et al., *Nucleic Acids Research*, 2020; Huuki-Myers et al., *Science*, 2024). Even when we have used these approaches, an experienced expert must still generate annotations on which the model can be trained, so we argue that it is difficult to avoid user bias in assigning cell types based on imaging data. Importantly, to ensure reproducibility, we provide all our HALO settings files and raw images so that results can be easily reproduced or reprocessed by others. We thus also added the following sentence at the end of the same methods section:

Detailed information on the parameters used for segmentation, phenotyping, and quantification by the HALO algorithms can be accessed through the HALO settings files, which are available on GitHub at <https://github.com/LieberInstitute/Human_DLPFC_Deconvolution/tree/main/raw-data/HALO/settings_files> [[71]](https://sciwheel.com/work/citation?ids=16003061&pre=&suf=&sa=0). Output files with additional cell measurements are also located on GitHub <https://github.com/LieberInstitute/Human_DLPFC_Deconvolution/tree/main/raw-data/HALO> [[71]](https://sciwheel.com/work/citation?ids=16003061&pre=&suf=&sa=0). These files can be used to reprocess or reproduce our segmentation results.

**4. Similarly, in the same section, in PDF page 29, Line 9, "The same thresholds were applied to a given cell type across all tissue samples, regardless of the donors, including the size thresholds for nucleus and cytoplasm." The use of same thresholds for nucleus and cytoplasm across all tissue samples, regardless of donor variations, might not account for biological differences between samples. This could lead to inaccurate cell type quantifications.**

We understand the reviewer’s concerns and appreciate that there are variations in cell types across donors. In HALO, we were measuring the expression of known cell type specific marker genes, which are robustly expressed and show less variation. Furthermore, the use of the same thresholds for nuclear and cytoplasmic segmentations across donors was done for the reasons described above to be consistent in our analysis. Based on our evaluation of segmentation accuracy during algorithm development, the thresholds differentiate cell types accurately across donors and we are able to detect biological differences between samples (**Rebuttal Fig 1**). For example, the Br6432_post sample has more OligoOPC than Br8667_mid sample (0.255 and 0.021 respectively) and we are able to detect that variation in the RNAscope/IF data (**Fig S10C’**, 1st and 7th rows). We also highlight, as mentioned above, that we are using a range of values for the nucleus that allows for some variation while excluding nuclear fragments and potential aggregates.

In addition, in the RNAScope/IF data we segmented a range of 28k-57k nuclei from each tissue section for a total of 1,045,597 total nuclei (median = 40,035 nuclei). Compared to the snRNA-seq which contains a total of 56k nuclei (median sample = 2,928) we have captured measurements from 18 times more cells. Thus we are actually better powered in the RNAscope/IF to detect variability.

As stated in the previous response, despite some limitations, we have found HALO to be an effective tool for nuclear segmentation and identification of broad cell types labeled by robust marker genes. Should a reader wish to mine our reference dataset, we have made images and HALO settings available for re-analysis if more complex cell type qualifications are needed.

**Rebuttal Fig 1. Example fluorescence image & HALO segmentation** **A.** Representative field from an smFISH experiment where SLC17A7, GAD1, MBP, and AKT3 were used. **B.** Segmentation performed in HALO, which is the segmentation of all applied user developed phenotype algorithms, providing accurate cell type calling

**5. In the Section "Evaluation of Deconvolution Results" PDF page 32, line 39, the use of the Pearson correlation as evaluation of the method has several limitations, such as it assumed linear relationship, normality etc. Other methods of evaluation such as Concordance between the groups, or Spearman's rank correlation coefficient would have been more appropriate. These alternatives can offer a more nuanced and robust evaluation of deconvolution results, leading to more reliable conclusions.**

We agree that evaluating Spearman's correlation would add to our evaluation of the methods. We have calculated Spearman’s correlation and added a supplemental figure and results to the manuscript.

With Spearman’s correlation, for the MeanRatio_top25 set of marker genes, *Bisque* consistently has the top correlation across all library types and RNA extractions (**Fig S19A**). *hspe* is still the second best performing method (in all but RiboZeroGold_Nuc), but with a larger gap than when evaluated with pearson correlation (**Fig 4B**). With Spearman’s correlation *CIBERSORTx* in polyA library types (**Fig S19A**) is much lower compared to Pearson’s correlation (**Fig 4B**), while in RiboZero samples *CIBERSORTx* is more similar to *hspe* and *Bisque*. *DWLS*, *BayesPrism*, and *MuSiC* are still the bottom three methods with poor Spearman’s correlation to the RNAScope data (**Fig S19A**).

Considering the sets of marker genes we tested, with *Bisque* the FULL marker set has slightly higher Spearman’s correlation than Pearson’s, the opposite is true of MeanRatio_top25, the difference is very minor and either marker set yields better correlation with the RNAScope proportions than other methods or marker sets (**Fig S19B**).

Using Spearman's instead of Pearson’s correlation does not change the main conclusion of *Bisque* and *hspe* as the top methods, nor the conclusions on marker gene selection as this is method dependent.

We have made the following changes to the manuscript:

Text added to Results: *Benchmark of Selected Deconvolution Methods with Mean Ratio top25 Marker Genes*

Alternatively, evaluating the proportion predictions with Spearman’s correlation ranked *Bisque* as the top method across all library types and RNA Extractions ([**Fig S**](https://docs.google.com/document/d/1Rk5Z8iyekZoVaYxqjl4nIHx9wTo4p8GjR1J1vki-9Ow/edit#sfi_spearman_cor)**19A**). Overall the ranking of deconvolution methods was similar across the two correlation measures.

*Results: Benchmark of Deconvolution Methods with Different Gene Sets*

These results are overall robust to the choice of correlation estimator (**Fig S19B**).

New Supplementary Figure:

[**Fig S**](https://docs.google.com/document/d/1Rk5Z8iyekZoVaYxqjl4nIHx9wTo4p8GjR1J1vki-9Ow/edit#sfigu_spearman_cor)**19:** **Deconvolution method performance evaluated with Spearman Correlation. A.** Spearman's correlation (cor_spearman) between the predicted proportions by deconvolution methods with MeanRatio_top25 marker genes and the estimated RNAScope/IF proportions across RNA extraction method and library type combinations, point size reflects the rmse value. **B.** Scatter plot of overall pearson correlation (cor) compared to Spearman’s correlations (cor_spearman) for each method (color) and set of marker genes (point shape).

**Fig 4B.** Correlation (cor) between the predicted proportions by deconvolution methods and the estimated RNAScope/IF proportions across RNA extraction method and library type combinations, point size reflects the rmse value. (Same as manuscript, added for reference)

We have also added this point to the discussion:

Consistent with other independent benchmark findings using human brain data ([**Table 1**](https://docs.google.com/document/d/1Rk5Z8iyekZoVaYxqjl4nIHx9wTo4p8GjR1J1vki-9Ow/edit#tab_methods)) [[17]](https://sciwheel.com/work/citation?ids=14539797&pre=&suf=&sa=0), we identified *Bisque* and *hspe* (an update on *dtangle* [[44]](https://sciwheel.com/work/citation?ids=6017166&pre=&suf=&sa=0)) as the most accurate deconvolution algorithms and showed that they are robust to RNA-seq library types, and RNA extractions, or choice of correlation method for evaluating the results ([**Fig S**](https://docs.google.com/document/d/1Rk5Z8iyekZoVaYxqjl4nIHx9wTo4p8GjR1J1vki-9Ow/edit#sfi_spearman_cor)**19**).

**Conclusion:**

**In summary, while the paper addresses a relevant topic and presents a quality dataset of the postmortem human prefrontal cortex, it does not provide a novel deconvolution approach. The Mean Ratio method has some fundamental issues which are mentioned in the detailed comments above. Significant revisions are necessary to improve its methodological rigor and clarity.**

We again thank the reviewer for their time and input in evaluating our manuscript. We have made edits to the manuscript as well as the code documentation of *DeconvoBuddies* to provide more detail into our recommendations for users of *DeconvoBuddies* that aim to help them use our software only when it’s appropriate to do so. The edits to the manuscript are aimed at improving the presentation of the properties of the benchmarking dataset we generated and the robustness of our conclusions.

# Reviewer: 3

**This manuscript presents a comprehensive benchmark of six bulk RNA-seq deconvolution methods using a novel dataset specifically generated for this benchmark. The dataset comprises snRNA-seq, RNAScope/IF, Visium, and bulk RNA-seq from 22 tissue blocks, utilizing different RNA extraction protocols. Previous benchmark studies have been inconclusive, likely due to variations in study design, including differences in the characteristics of reference scRNA-seq datasets, marker gene selection, and simulation approaches. While this benchmark may not address all questions, it provides valuable insights and the authors are to be commended for their diligent work. Independent benchmarks are often particularly valuable for the field, as are reference datasets with a carefully derived "gold standard".**

**I have a few comments that will hopefully help to further improve the manuscript. The first comment addresses what I believe is an important question that has not been fully covered in the benchmark study. The additional comments are either for clarification or pertain to less important issues.**

We would like to thank the reviewer for their time reading our manuscript and providing comments. Accordingly, we have revised the manuscript to help clarify these points to our future readers. We hope that these changes will help improve the readability and clarity of the manuscript.

**1) Towards the end of the manuscript, the authors assess the performance of Bisque and hspe when non-matched external snRNA-seq reference data from the same brain region (DLPFC) is used. They speculate that the smaller number of nuclei in the Tran et al. reference may contribute to Bisque's poorer performance with this reference, but also note the different cell type composition of this dataset. I believe this is a highly relevant question that warrants further investigation: How large should an snRNA-seq reference dataset be to achieve good deconvolution results? There are much larger DLPFC reference datasets with over 1.5 million nuclei (Kellis/Tsai Cell paper and the De Jager Nature Genetics paper) that could be used to evaluate the methods' performances with increasing reference dataset size. These datasets should be large enough to observe convergence well before the entire dataset is used.**

We agree with the reviewer that this analysis of increasing the number of donors will strengthen our exploration of reference set qualities. However, the two suggested datasets were not a good fit for our purposes here as Pindea et al., 2024 (from the Kellis group) only has 16 control donors, and Fujita et al., 2024 (from the De Jagar group) is ROSMAP data from advanced age individuals that are older than our samples (median age = 50.3). Instead, we chose to use the CommonMind (CMC) snRNA-seq dataset from the PsychENCODE Consortium, with 52 control samples and 254k nuclei (Emani et al., *Science*, 2024).

We found Bisque out performed hspe in this dataset regardless of number of donors used, but the median performance metrics were relatively flat across number of donors. We did find that the performance of Bisque stabilized with an increasing number of donors. This analysis helps further understand how both methods perform on different sized datasets.

We have added the following text to the methods and results sections, as well as a new Supplementary Figure.

Methods sub-section “Donor subsampling simulation”:

To evaluate the impact of donor quantity on deconvolution results, deconvolution was performed using *Bisque* and *hspe* (*Methods: Deconvolution)* on various subsets of donors. The 52 neurotypical control donors from the CMC data were used (Emani et al. 2024; (PEC) 2023), with the .var[‘subclass’] column of the AnnData object matched to broad cell-type resolution, which involved dropping Immune, PC, and SMC cells. *Mean Ratio top25* markers were computed and filtered to ensure ratios exceeded 1; this resulted in 25 markers for each cell type with the exception of EndoMural cells, which had 19 markers. Next, data was downsampled to include various numbers of donors between three and the full set of 52. For each of these ten fixed numbers of donors, 100 iterations of deconvolution with both *Bisque* and *hspe* were performed, each selecting a different random combination of donors to control for possible selection bias.

New Supplementary Figure:

[**Fig S3**](https://docs.google.com/document/d/1Rk5Z8iyekZoVaYxqjl4nIHx9wTo4p8GjR1J1vki-9Ow/edit#sfigu_cmc_donor_subset)**1**: **Performance of Bisque and hspe on subsets of donors from the CMC DLPFC snRNA-seq dataset. A.** Scatter plot of cell type proportions estimated by RNAScope/IF (x-axis) vs. the mean predicted cell type proportions by the deconvolution methods over 100 replicates at different subsets of the total donors. Proportions were calculated with the Mean Ratio top25 marker set. Points are colored by cell type and shaped by the combination of the bulk RNA-seq sample’s library type and RNA extraction. Pearson correlation (cor) and root mean squared error (rsme) values are shown for each panel. Boxplots of **B.** Pearson correlation (cor) and **C.** RMSE of predicted proportions vs. RNAScope/IF proportions for the 100 subsets of an increasing number of donors.

Results sub-section: Benchmark of Deconvolution Methods with Other Datasets

To expand on the performance of these methods on increasingly large datasets, we utilized the PsychENCODE Common Mind Consortium DLPFC snRNA-seq dataset to test accuracy of these methods from 3 to all 52 donors (Emani et al. 2024; (PEC) 2023). With this input dataset, *Bisque’s* mean proportions across iterations have higher correlation with the RNAScope/IF data than *hspe,* across all tested number of donors ([**Fig S3**](https://docs.google.com/document/d/1Rk5Z8iyekZoVaYxqjl4nIHx9wTo4p8GjR1J1vki-9Ow/edit#sfi_cmc_donor_subset)**1A**). Observing performance in individual subset runs, *Bisque* shows large improvements in consistency with increasing number of donors, *hspe* shows some improvements with more donors has much more variable performance. For both methods the median cor and rmse values are relatively flat across increasing donors ([**Fig S3**](https://docs.google.com/document/d/1Rk5Z8iyekZoVaYxqjl4nIHx9wTo4p8GjR1J1vki-9Ow/edit#sfi_cmc_donor_subset)**1B-C**).

**2) Regarding the RNAscope/IF cell type proportions, it is unclear why so many cells are classified as "other." For instance, in Fig 2D sample BR8667_mid has about 50% of cells classified as Ast/Mic/Exc/Inh/Oli/End and the other half as "other cells." Does this reflect high uncertainty in the RNAscope/IF-based cell type classification? This should be explained in more detail. Considering these results, the authors should also discuss why they prefer RNAscope/IF as the "gold standard" over snRNA-seq. It is not obvious to me which of these two methods yields more accurate cell type proportion estimations, as both have potential biases. Perhaps it would be more appropriate to consider both references equally.**

The large proportion of “other” cells is expected and due to the multiplexing limitations of the RNAScope/IF assay. As this assay is limited to four probes, we could only measure three cell types in a single experiment as one probe was used to measure a proxy for total RNA expression with *AKT3*. This is why we used a two combination experimental design to cover six major cell types across two consecutive tissue slides (**Fig2A-B**). In the “Circle” combination, we labeled Astro, EndoMural, and Inhib cell types, therefore “Other” cells would include Excit, Micro, and OligoOPC, which were captured in the adjacent tissue slice in the “Star” combination (**Fig 2A**). In addition, rare cell types that were not targeted in our panel, such as immune cells, would contribute a small proportion to the “Other” category as well.

To make this more clear to readers we’ve added a sentence to the RNAScope/IF results discussing the measured “Other” proportions.

Results: Orthogonal Cell Type Proportion Measurement with RNAScope/IF Imaging

In each combination, a large proportion of “Other” cells were observed. This is expected as we only probe for 3 of the 6 broad cell types in each assay due to multiplexing limitations with RNAscope/IF.

In relation to which assay (RNAScope/IF or snRNA-seq) provides better estimates of cell composition, in addition to **Fig S17**, we would like to refer to our responses to Reviewer 2, question 4 (further above). Briefly, RNAScope/IF is an assay that measures a larger number of cells than snRNA-seq (~1 million vs ~56,000 cells; Reviewer 2, question 4), thus theoretically providing a more detailed picture. This doesn’t mean that RNAScope/IF is perfect, but we did find RNAScope/IF to be the better cell type proportion reference to compare against.

**3) Why do all methods demonstrate poor estimations for astrocytes? At the same time, the RNAscope-derived astrocyte proportions seem overestimated for some samples. Can the authors speculate on why the methods don't work well for astrocytes, or whether this may be caused by the reference?**

Given the complex morphology of astrocytes and diffuse staining of GFAP antibody, this cell type was one of the most difficult to segment in RNAscope/IF analyses. In many tissue sections, especially those with larger proportions of white matter, we observed widespread and high intensity signal of astrocytic processes. When developing phenotype algorithms it was necessary to balance the detection of false positive and negative signals such that nuclei with astrocytic labeling were true astrocytes and not neighboring processes. We also note that samples with the highest proportion of astrocytes also have the highest proportion of white matter (from Visium spatial transcriptomics experiments in Huuki-Myers et al., *Science*, 2024), reflecting that these samples have larger white matter regions compared to other samples.

More specifically, the estimated Astrocyte proportions are positively correlated with the amount of white matter predicted by Visium spatial transcriptomics from the same tissue block. As noted above, this correlation is stronger than the snRNA-seq proportions (**Rebuttal Fig 2**).

However, to further investigate the impact of the inclusion of astrocytes in the conclusions of our work, we repeated **Figure 4B** without astrocytes and added a new supplementary figure and related results to the manuscript. We found that the ranking of the deconvolution methods is consistent with or without the inclusion of astrocytes.

**Rebuttal Fig 2.** Scatter plot of proportion of tissue block annotated as white matter (WM) in Visium spatial transcriptomics compared to proportion of Astrocytes measured by RNAscope/IF and snRNA-seq. Annotation shows Pearson correlation value.

[**Fig S**](https://docs.google.com/document/d/1Rk5Z8iyekZoVaYxqjl4nIHx9wTo4p8GjR1J1vki-9Ow/edit#sfigu_metrics_no_astro)**20.** **Deconvolution method performance evaluated without Astro. A.** Pearson correlation (cor) between the predicted proportions by deconvolution methods with MeanRatio_top25 marker genes and the estimated RNAScope/IF proportions across RNA extraction method and library type combinations, excluding Astro predictions, point size reflects the rmse value (related to [**Figure 4**](https://docs.google.com/document/d/1Rk5Z8iyekZoVaYxqjl4nIHx9wTo4p8GjR1J1vki-9Ow/edit#fig_benchmark)**B**). **B.** Scatter plot of Pearson correlation (cor) and RMSE with all cell types and without Astro, across all samples (GLOBAL) and across RNA extraction method and library type combinations (point shape).

### Benchmark of Selected Deconvolution Methods with *Mean Ratio top25* Marker Genes

Given the discrepancy between RNAScope/IF and snRNA-seq proportions for astrocytes (**Fig 2E**), likely due to the difficulty of segmenting astrocytes due to their complex morphology, we evaluated deconvolution results without astrocytes. We found that correlation of all methods improves against RNAScope/IF and the overall ranking of methods is mostly invariant, with the exception of *hspe* slightly outperforming *Bisque* (**Fig S20**).

**4) The benchmark demonstrates the sensitivity of some methods to the selection of marker genes. I agree with the approach of comparing all methods using the same set of marker genes, as performed in the present study. However, it would also be beneficial to run each method once using the exact marker gene selection procedure suggested by the authors of the respective methods (as listed in Table 1) and report those results either in Table 1 or in the supplement.**

We agree with the reviewer, and this was completed with the “FULL” marker gene set. The full dataset was fed into the each method pipeline, where some methods (*MuSiC*, *BayesPrism*, *hspe*, and *CIBERSORTx*) apply their own marker gene selection/weighting. To make this more apparent to readers we clarified this in the text in the results and methods sections:

“Deconvolution methods were also run with the “Full” set of common genes between the snRNA-seq and bulk RNAseq data (defaulting to each methods built-in marker selection if present)”

“Five sets of gene were selected to benchmark in the deconvolution methods:

1. *Full*: set of genes common between the bulk and snRNA-seq datasets (17,804 genes). Equivalent to the default marker selection for each method”

**5) Can the authors clarify whether section BR8325_mid has a very large fraction of WM, or if it is an outlier that results in an overestimation of oligodendrocytes by all methods?**

We examined samples from tissue block Br8325_mid more closely to help explain the high estimation of Oligo or OPC across methods.

In the related Visium spatial resolved transcriptomic data from Hyuuki-Myers et al., 2024, in k=2 BayesSpace clustering (which separates white matter and gray matter), Br8325_mid is predicted to be 90.2% gray matter and 9.8% white matter, close to the median values 89.9% gray matter, 10.1% white matter in the 19 tissue sections with both data types (**Rebuttal Fig 3**). This shows that Br8325_mid has a typical if not slightly lower proportion of WM, which would not explain the high estimations of Oligo or OPC.

**Rebuttal Fig 3. Proportion of White Matter by tissue block.** Estimated by proportion of spots in paired Visium spatial transcriptomics dataset (**Fig 1A**) (Huuki-Myers et al., 2024).

In the RNAScope/IF data, Br8325_mid is predicted to have a proportion of OligoOPC of 0.18, compared to the median of 0.12. In snRNA-seq the proportion of Oligo is 0.24 slightly higher than the median of 0.17. Neither of these cell types estimates explain the high Oligo or OPC estimates.

This tissue section also does not appear to be an outlier in bulk sampling quality control metrics either (**Rebuttal Fig 4**). One sample Br8325_mid_polyA_Nuc, flagged as “qc_warn”, has low concordance_map_rate, overallMapRate. However the other Br8325_mid samples with normal qc metrics all show the same high oligo pattern, other qc_warn samples from other tissue blocks don’t have abnormal Oligo predictions. Issues of quality in Bulk RNA-seq do not seem to drive this prediction either.

**Rebuttal Fig 4. Quality Control metrics by tissue block.** Br8325_mid is highlighted in red.

Examining the Principal components of the bulk samples, section Br8325_mid is an outlier in PC4 (**Rebuttal Fig 5A**). We examined the correlation between the bulk PCs and phenotype and quality metrics to learn more about what could drive this signal, PC4 is most strongly correlated with donor (BrNum), the other tissue block from the same donor (Br8325_ant) does not show the same high Oligo pattern (**Rebuttal Fig 5B, Fig S16**). PC4 shows us that something is different about the gene expression in this tissue block, but the source of this difference is still unknown.

To ensure the results of our benchmark are not skewed by the bulk samples from Br8325_mid, we recomputed the correlation and rmse values for the deconvolution methods excluding these samples. The results are largely unchanged, the correlation in the top methods improves slightly, and rmse drops for almost all methods (**Rebuttal Fig 5C-D**).

While we were not able to identify the factor driving the high estimation of Oligo or OPC from all methods, this difference in the tissue block Br8325_mid did not impact our conclusions about deconvolution method performance.

**Rebuttal Fig 5. Principal component of bulk RNA seq and benchmark results excluding outlier Br8325_mid. A.** Boxplots of PC4 in build RNA-seq samples by tissue block, Br8325_mid is highlighted in blue. **B.** Heatmap of Canonical Correlation Analysis (from *VariancePartition* v1.34.0 canCorPairs and plotCorMatrix) of top 5 PCs, phenotype data, and quality control metrics in the bulk RNA-seq data. **C.** Pearson correlation (cor) between the predicted proportions by deconvolution methods with MeanRatio_top25 marker genes and the estimated RNAScope/IF proportions across RNA extraction method and library type combinations, excluding Br8325_mid, point size reflects the rmse value (related to [**Figure 4**](https://docs.google.com/document/d/1Rk5Z8iyekZoVaYxqjl4nIHx9wTo4p8GjR1J1vki-9Ow/edit#fig_benchmark)**B**). **D.** Scatter plot of pearson correlation (cor) and RMSE with all samples vs. excluding Br8325_mid, across all samples (GLOBAL) and across RNA extraction method and library type combinations (point shape).

**6) The laminar structure of the cortex can vary considerably. The sections used in this study to generate the bulk RNA-seq, RNAscope, and snRNA-seq data were sandwiched between two Visium sections (Fig 1A). It is fairly easy to detect WM/GM using Visium ST data. Have the authors considered estimating the proportion of WM at both ends of the tissue block using their existing Visium data to determine how much of the cell type proportion errors may be attributable to changes in the laminar structure along the tissue block?**

We thank the reviewer for this excellent suggestion and agree that slight changes in proportion are expected as cryosections are taken across the anterior/posterior access of the PFC. However, based on our previous Visium analyses, we expect that these shifts will be small.

For 4 out of the 30 tissue blocks, we have Visium and Visium-SPG data at the furthest ends of the tissue blocks. The fluctuation in the proportion of white matter spots between Visium and Visium-SPG provides an estimate on the expected variation of WM across our tissue blocks (**Rebuttal Fig 6**), which is limited. The morphology of the tissue slices from the ends of the block is also visually similar (**Rebuttal Fig 7**), supporting that changes between slices within the assays of this datasets should be small.

**Rebuttal Fig 6.** **Scatter plot of the proportion of white matter (WM) spots from Visium vs. Visium-SPG taken from the same tissue block.** As noted in **Fig S1**, Visium and Visium-SPG (formerly Visium-IF) are the opposite ends of the tissue blocks for which they were generated. Blue line is the linear fit of the data, black line is x=y.

**Rebuttal Fig 7**. **White matter in tissue block Br6432_ant**. **A.** Spot plot of k=2 clustering in Visium, the light spots denote white matter (Sp2D1~WM) dark spots are gray matter (Sp2D2~GM). **B.** Spot plot of WM vs. GM annotations in Visium-SPG. **C.** Immunofluorescence image from the VIsium-SPG section.

**7) Finally, I would like to encourage the authors to prepare a Bioconductor data package including the processed bulk RNA-seq and snRNA-seq data, as well as the results (cell type proportions) from the RNAscope experiments. Such a package would further facilitate the use of this benchmark dataset in future studies.**

We agree with the reviewer and have further improved *DeconvoBuddies* to the point that we were able to submit it to Bioconductor. As noted at <https://github.com/LieberInstitute/DeconvoBuddies/actions/workflows/check-bioc.yml>, *DeconvoBuddies* passes R CMD check and BiocCheck on linux and Windows using Bioconductor version 3.20 (current bioc-devel). The revision of the package at Bioconductor is currently ongoing at <https://github.com/Bioconductor/Contributions/issues/3503>.

In more detail, <https://research.libd.org/DeconvoBuddies/reference/fetch_deconvo_data.html> provides access to the processed bulk and snRNA-seq data. The RNAScope/IF cell type proportions are included as data in the package and documented at <https://research.libd.org/DeconvoBuddies/reference/RNAScope_prop.html>.

**Second round of review**

**Reviewer 1**

This manuscript undertakes a comparative analysis of various cellular deconvolution methods applied to gene sets from postmortem human prefrontal cortex (DLPFC) samples. The authors have addressed previous concerns to some extent; however, further improvements are still necessary. These improvements should focus on methodological detail, analytical depth, and clarity of presentation. Below, I will outline specific details for each point.

Key Concerns:
1, The manuscript tests six deconvolution methods using different top gene sets, including 1vALL top25 (number of genes = 145), Mean Ratio top25 (number of genes = 151), Mean Ratio MAD3 (number of genes = 520), Mean Ratio over2 (number of genes = 557), and a full set of common genes (number of genes = 17,804). Notably, only the full gene set shows variation in performance metrics, as depicted in Fig. S26. It seems that only the results from the two extremes of selecting few gene with large signals and the whole set of genes are shown. In typical analyses, a few thousand highly variable genes are often considered. It would be great if the authors can present more result with a few thousand highly variable genes selected. Furthermore, the use of only two metrics to evaluate the methods seems insufficient for a benchmark study. A broader range of measures, at both the sample and cell type levels, would offer a more comprehensive assessment of method performance.

2, As a benchmarking study, it would be beneficial for the authors to evaluate the performance of cell-type deconvolution methods across different experimental platforms, including snRNA-seq and scRNA-seq technologies, which are pertinent to the study’s title. Additionally, examining the computational efficiency of each method and providing guidance for method selection based on specific research interests and dataset characteristics would greatly benefit readers.

3, The new Mean Ratio method introduced requires further validation. It is necessary to test its performance using marker genes selected by the proposed method and compare these results with outcomes using traditional marker selection methods. This comparative analysis is essential to substantiate the method’s advantages or limitations. Additionally, the performance of base methods should be compared using both the recommended marker gene selection criteria and the marker genes selected by the Mean Ratio method to provide a comprehensive assessment of its relative efficacy.

Minor Points:
1, The performance of the methods should be evaluated when the reference dataset does not match the bulk tissue from the DLPFC. This information is critical for assessing method applicability.

2, Biological insights derived from using base deconvolution methods to estimate cell type proportions in the DLPFC should be discussed. The manuscript should also consider the potential for identifying more refined cell types, beyond the six major cell types measured by orthogonal RNAScope/IF dataset, to evaluate the methods' effectiveness in heterogeneous tissues.

3, Inconsistencies in figure legends, such as those seen in Fig. S26 and Fig. S4, should be corrected. For example, the titles for the y-axis are missing in Fig.S4. Ensuring clarity and consistency in figure legends is necessary to facilitate clear understanding of the data.

**Reviewer 2**

The authors have addressed my concerns.

**Reviewer 3**

The authors have addressed all my comments, and I believe this benchmark study provides valuable new insights.

However, I have one remaining concern regarding Supplemental Figure 26B, which was added in response to a comment from the first reviewer. The reviewer raised a valid point that the number of marker genes could impact the methods’ performance. One might expect a non-linear relationship, as beyond a certain point, adding more genes may introduce noise without much additional information. Using a linear regression line based on only four data points as in the figure does not seem adequate to address the reviewer’s question. More data should be presented.

**Authors Response**

**Point-by-point responses to the reviewers’ comments:**

Please find below our detailed response to Reviewers. Reviewer comments are in black and responses are in blue. In the revised manuscript, we have highlighted all new text in red.

# Reviewer 1

This manuscript undertakes a comparative analysis of various cellular deconvolution methods applied to gene sets from postmortem human prefrontal cortex (DLPFC) samples. The authors have addressed previous concerns to some extent; however, further improvements are still necessary. These improvements should focus on methodological detail, analytical depth, and clarity of presentation. Below, I will outline specific details for each point.

**Key Concerns:**

**1.** The manuscript tests six deconvolution methods using different top gene sets, including 1vALL top25 (number of genes = 145), Mean Ratio top25 (number of genes = 151), Mean Ratio MAD3 (number of genes = 520), Mean Ratio over2 (number of genes = 557), and a full set of common genes (number of genes = 17,804). Notably, only the full gene set shows variation in performance metrics, as depicted in Fig. S26. It seems that only the results from the two extremes of selecting few gene with large signals and the whole set of genes are shown. In typical analyses, a few thousand **highly variable genes** are often considered. **It would be great if the authors can present more result with a few thousand highly variable genes selected.**

We agree with the reviewer that the analysis of performance vs. number of genes could be strengthened by including more data points. We have applied the reviewer's idea, and tested 10 to 100% (at 10% increments) of highly variable genes as selected by scran::modelGeneVar in the snRNA-seq data, subset to the common set of genes. This resulted in sets of genes from 811 to 8113 genes. The deconvolution methods were run on these gene sets and their performance was assessed with Pearson's correlation & RMSE. We have added a new methods section *“Highly Variable Gene Sets”*, and edited “*Methods: Deconvolution*” in the manuscript documenting this process.

This HVG analysis mostly confirms trends we had already observed:

- *Bisque*, *hspe*, and *CIBERSORTx* are the top performing methods
  - *Bisque* best balances rmse & cor and is most stable across genesets
  - *hspe* has the lowest rmse values
  - *CIBERSORTx* has the highest cor (*Bisque* close behind)
- *hspe* and *CIBERSORTx* performance is sensitive to gene sets
  - selecting smaller sets of variable or marker genes improves *hspe*
  - less clear pattern from *CIBERSORTx*
- *MeanRatio_top25* (light green) has best balance of cor & rmse for all top methods

In terms of predicting performance of number of genes, the additional HVG sets show that most methods cannot linearly fit correlation or rmse to number of genes, only *BayesPrism* had a statistically significant linear fit (p<0.05) for pearson’s correlation, and *hspe* for RMSE. Other methods were either stable or unpredictable as the number of genes changed.

We have added the results of the HVG analysis to Fig 5, an additional supplementary figure Fig S23, and updated Fig S26.

We have also updated the following text:

*Results: Benchmark of Deconvolution Methods with Different Gene Sets*

We also tested sets of 10% to 100% of Highly Variable Genes (HVGs) in the snRNA-seq data (*Methods: Highly Variable Genes*).

Deconvolution methods had varying and unpredictable performance when using different sets of genes: marker genes ([**Figure 5**](https://docs.google.com/document/d/1Rk5Z8iyekZoVaYxqjl4nIHx9wTo4p8GjR1J1vki-9Ow/edit#fig_benchmark_marker)) and increasing HVGs sets ([**Fig S23**](https://docs.google.com/document/d/1Rk5Z8iyekZoVaYxqjl4nIHx9wTo4p8GjR1J1vki-9Ow/edit#sfi_cor_rmse_scater_overall)). Broadly, *Bisque*, *hspe*, and *CIBERSORTx* had the top correlation values compared to the RNAScope/IF proportions across the different marker sets. *Bisque* was the most stable across the different marker gene sets ([**Figure 5**](https://docs.google.com/document/d/1Rk5Z8iyekZoVaYxqjl4nIHx9wTo4p8GjR1J1vki-9Ow/edit#fig_benchmark_marker)). For *Bisque* the top correlation was with *MeanRatio top25* (cor = 0.538) and the lowest marker gene set was *MeanRatio over2* (cor = 0.504, [**Figure 5**](https://docs.google.com/document/d/1Rk5Z8iyekZoVaYxqjl4nIHx9wTo4p8GjR1J1vki-9Ow/edit#fig_benchmark_marker)**A**, [**Fig S24**](https://docs.google.com/document/d/1Rk5Z8iyekZoVaYxqjl4nIHx9wTo4p8GjR1J1vki-9Ow/edit#sfi_prop_scater_MR), [**Fig S25**](https://docs.google.com/document/d/1Rk5Z8iyekZoVaYxqjl4nIHx9wTo4p8GjR1J1vki-9Ow/edit#sfi_ggprop_marker_bisque)) with the lowest overall input set being HVG70 (cor = 0.504, [**Fig S23**](https://docs.google.com/document/d/1Rk5Z8iyekZoVaYxqjl4nIHx9wTo4p8GjR1J1vki-9Ow/edit#sfi_cor_rmse_scater_overall)). *hspe* was more sensitive to marker selection as evident by the range of correlation when compared to RNAScope/IF proportions with different gene set inputs (max cor: 0.596 with *MeanRatio over2*, min cor:-0.11 with HVG100, [**Figure 5**](https://docs.google.com/document/d/1Rk5Z8iyekZoVaYxqjl4nIHx9wTo4p8GjR1J1vki-9Ow/edit#fig_benchmark_marker)**A**, [**Fig S23**](https://docs.google.com/document/d/1Rk5Z8iyekZoVaYxqjl4nIHx9wTo4p8GjR1J1vki-9Ow/edit#sfi_cor_rmse_scater_overall), [**Fig S24**](https://docs.google.com/document/d/1Rk5Z8iyekZoVaYxqjl4nIHx9wTo4p8GjR1J1vki-9Ow/edit#sfi_prop_scater_MR), [**Fig S26**](https://docs.google.com/document/d/1Rk5Z8iyekZoVaYxqjl4nIHx9wTo4p8GjR1J1vki-9Ow/edit#sfi_ggprop_marker_hspe)). The performance of *BayesPrism* substantially improved with the Full marker set ([**Figure 5**](https://docs.google.com/document/d/1Rk5Z8iyekZoVaYxqjl4nIHx9wTo4p8GjR1J1vki-9Ow/edit#fig_benchmark_marker)**A**, [**Fig S23**](https://docs.google.com/document/d/1Rk5Z8iyekZoVaYxqjl4nIHx9wTo4p8GjR1J1vki-9Ow/edit#sfi_cor_rmse_scater_overall), [**Fig S24**](https://docs.google.com/document/d/1Rk5Z8iyekZoVaYxqjl4nIHx9wTo4p8GjR1J1vki-9Ow/edit#sfi_prop_scater_MR)). The best choice of marker gene set is dependent on the selected deconvolution method, for example *MuSiC* as evaluated by correlation is sensitive to the set of marker genes with *1vAll top25* producing the most accurate results. For most methods, the relationship between number of marker genes and correlation or rmse is not linear ([**Fig S27**](https://docs.google.com/document/d/1Rk5Z8iyekZoVaYxqjl4nIHx9wTo4p8GjR1J1vki-9Ow/edit#sfi_accuracy_n_marker)), and accuracy cannot be simply predicted from the number of genes. Overall, *Bisque* was the most stable when correlation and rmse were jointly considered across library types and RNA extractions ([**Figure 5**](https://docs.google.com/document/d/1Rk5Z8iyekZoVaYxqjl4nIHx9wTo4p8GjR1J1vki-9Ow/edit#fig_benchmark_marker)**B**). These results are overall robust to the choice of correlation estimator ([**Fig S19**](https://docs.google.com/document/d/1Rk5Z8iyekZoVaYxqjl4nIHx9wTo4p8GjR1J1vki-9Ow/edit#sfi_spearman_cor)**B**).

**Fig S23. Deconvolution method performance with various marker or gene sets.** Scatter Plot between the overall cor and rmse values for cell type proportion predictions for each method (shape) and gene set (point color). Related to [**Figure 5**](https://docs.google.com/document/d/1Rk5Z8iyekZoVaYxqjl4nIHx9wTo4p8GjR1J1vki-9Ow/edit#fig_benchmark_marker).

**Fig S27. Linear fit of number of marker genes and quality metric values for cell type proportion predictions.** Scatter plot of number genes on the x-axis compared to **A.** Pearson’s correlation (cor) and **B.** Root mean squared error (rmse) on y-axis for each deconvolution method (color). Adjusted R2 values are added as annotations, linear fits with p-value <0.05 are indicated with “*” (*BayesPrism* for cor, and *hspe* for rmse).

Furthermore, the use of only two metrics to evaluate the methods seems insufficient for a benchmark study. **A broader range of measures, at both the sample and cell type levels, would offer a more comprehensive assessment of method performance.**

Correlation and root mean square error (RMSE) are used as evaluation metrics in other similar benchmark studies; Dai et al., 2024 used Spearman’s correlation and RMSE, Cobos et al., 2020 used Pearson’s correlation and RMSE. In addition to RMSE and Pearson’s correlation, we also evaluated Spearman’s correlation at the previous request of reviewer 2 (Fig S19). Spearman’s correlation supported our ranking of the deconvolution methods. Overall, our approach has been consistent with previously published and cited benchmarks.

**2.** As a benchmarking study, it would be beneficial for the authors to evaluate the performance of cell-type deconvolution methods across **different experimental platforms, including snRNA-seq and scRNA-seq technologies, which are pertinent to the study's title**.

While we understand the reviewer’s point about testing across both snRNA-seq and scRNA-seq datasets, unfortunately, almost all postmortem human brain datasets are derived from fresh frozen tissue that generates single nuclei as opposed to single cells (as freezing the tissue lyses the cells). Therefore, there are not many available single cell datasets available from fresh human brain tissue, with the exception of such efforts as the Living Brain Project (<https://icahn.mssm.edu/research/friedman/living-brain>). In rare cases where existing single cell brain data is available, it is often obtained via a surgical resection from a disease state and with low sample sizes. With these factors in mind, researchers applying our findings to human brain data will likely be working with single nucleus references.

We also emphasize that previous work from Bakken et al. 2018 demonstrated that single nucleus RNA-seq data is representative of single cell RNA-seq (doi: 10.1371/journal.pone.0209648). Finally, the strength of our benchmark is the paired datasets, including the RNAScope cell type proportions in human DLPFC. Given the limitations discussed above, we could not locate any single cell RNA-seq DLPFC datasets that we could use as a reference set for deconvolution. As our current title suggests, this work’s scope is focused on human brain:

Benchmark of cellular deconvolution methods using a multi-assay dataset from postmortem human prefrontal cortex.

While we understand the reviewer’s desire for thoroughness, we believe that testing single nucleus vs. single cell reference human brain data is out of the scope of this work and has limited value to human brain applications.

Additionally, examining the **computational efficiency** of each method and providing guidance for method selection based on specific research interests and dataset characteristics would greatly benefit readers.

We agree with the reviewer that computational efficiency is an important consideration for researchers, as long runtimes or high memory requirements may make a method impractical or even unusable.

When re-running the methods with the HVG sets (811 to 8113 genes) we took care to log runtime and max memory usage of each method, deconvoluting our 110 bulk DLPFC samples with the 56k nuclei snRNA-seq reference set.

For memory demands *MuSiC* and *hspe* were the most efficient, running our data sets with less than 5G max vmem across the different number of genes input. *DWLS* and *BayesPrism* often required 32G or more of memory, more than a laptop can typically provide. Memory required for *Bisque* and *CIBERSORTx* depended on the number of genes used, at <5,000 genes and similar sized inputs these methods could be applied on a laptop.

We found most methods were quick to run, *Bisque* and *hspe* being the fastest taking only a few minutes. *CIBERSORTx*, *BayesPrism*, and *MuSiC* taking between 30 min to 1 hour, and *DWLS* was the slowest taking up to 6 hours depending on the number of genes used.

These results match the reported trends in runtime and memory usage from Cobos et al. 2020, (Supplementary Fig 5) where *Bisque* runs faster and less memory intensive than *MuSiC* and *DWLS*, with *DWLS* being the least efficient in both metrics.

There are other variables, like size of reference and bulk RNA-seq datasets, cell type resolution, or details of computing platforms that may also impact the efficiency of these methods. We did not explore the effect of these variables as computational efficiency is not the focus of this work.

Accuracy is a much more critical aspect of method selection, and in our benchmark we found large differences in the performance of the methods. If a method is returning inaccurate results, low memory and speedy runtime doesn’t matter. Fortunately the top performing methods, *Bisque* and *hspe* both have fast runtimes and low memory requirements, which should make them both accessible choices for researchers.

We have added the following text and a new Supplementary Figure to the manuscript:

*Results: Benchmark of Deconvolution Methods with Different Gene Sets*

In terms of computation efficiency for deconvoluting this data, *Bisque* was the fastest method running in only a few minutes, and *hspe* was the least memory intensive, requiring less than 5GB ([**Fig S28**](https://docs.google.com/document/d/1Rk5Z8iyekZoVaYxqjl4nIHx9wTo4p8GjR1J1vki-9Ow/edit#sfi_runtime_mem)).

*Discussion*

Fast runtimes and relatively low memory demands should make these methods (*hspe* and *Bisque*) both accessible choices for researchers (Fig S28).

**Fig S28.** **Method computational efficiency across gene sets.** Line plots of **A.** Maximum virtual memory (max vmem) in gigabytes (GB) and **B.** wall clock time in minutes used to run the six deconvolution methods (color) over different sets of Highly Variable Genes with the DLPFC snRNA-seq and bulk datasets. DWLS and BayesPrism required significantly more memory (32G or more) and runtime, with DWLS being the slowest and most memory intensive method.

**3.** The new **Mean Ratio method introduced requires further validation**. It is necessary to test its performance using marker genes selected by the proposed method and **compare these results with outcomes using traditional marker selection methods**. This comparative analysis is essential to substantiate the method's advantages or limitations.

We proposed the *Mean Ratio* method specifically to find genes useful for deconvolution. In this work we evaluated how the different sets of marker genes impacted the accuracy of deconvolution.

The “traditional” marker selection strategy we tested against was 1 vs. All differential expression method (*t*-test), implemented in the widely used *scran* Bioconductor package. Comparing the top sets of 25 genes for each cell type, 65 genes were common between the two methods (out of 145 1vALL, and 151 for MeanRatio). In Figure 3 we showed how top Mean Ratio genes have clean cell type specific expression. In *Results: Benchmark of Deconvolution Methods with Different Gene Sets*, Figure 5, and Fig S23-24, we showed that performance of deconvolution methods with different input gene sets is often unpredictable. In those same figures, for the top 3 deconvolution methods (*CIBERTSORTx*, *hspe*, and *Bisque*), MeanRatio markers outperformed 1vALL_top25 and other gene sets in terms of deconvoluted cell type proportions compared against the orthogonal measurements, balancing cor and rsme (Figure 5B). We have also now added a comparison against increasingly large sets of HVGs (Fig S23), though HVGs are not necessarily cell type marker genes.

While there are other uses for cell type marker genes (like cluster annotation), evaluating them here is out of the scope of this work. Overall, we feel confident in recommending the use of *MeanRatio* for selecting input gene sets for deconvolution.

Additionally, the **performance of base methods** should be compared using both the recommended marker gene selection criteria and the marker genes selected by the Mean Ratio method to provide a comprehensive assessment of its relative efficacy.

We addressed a similar concern from Reviewer 3 comment 4 in the first round of revisions.

The “FULL” set of genes is provided to each “base” deconvolution method, where *MuSiC*, *BayesPrism*, *hspe*, and *CIBERSORTx* apply their own gene selection or weighting strategy. The results of the FULL gene set vs. MeanRatio are shown in Figure 5.

We highlighted that the “FULL” gene set is equivalent to the base method in the previous edits to results and methods sections of the manuscript.

**Minor Points:**

**1.** The performance of the methods should be evaluated when the **reference dataset does not match the bulk tissue from the DLPFC**. This information is critical for assessing method applicability.

We evaluated the performance of the top methods with non-paired snRNA-seq data in *Results: Benchmark of Deconvolution Methods with Other Datasets* and Figure 6, utilizing publicly available snRNA-seq data from Human DLPFC (Tran et al., and Mathys et al. datasets). We believe this is the most common scenario for researchers, and addresses the concern of the reviewer to evaluate “reference dataset does not match the bulk tissue”.

Beyond that it is unclear what this reviewer is suggesting. If the reviewer was suggesting to test performance with non-DLPFC snRNA-seq data, a reference dataset from a different brain region than the bulk tissue will always have inaccurate results due to differences in cell type populations between brain regions.

**2.** Biological insights derived from using base deconvolution methods to estimate cell type proportions in the DLPFC should be discussed. The manuscript should also consider the **potential for identifying more refined cell types**, beyond the six major cell types measured by orthogonal RNAScope/IF dataset, to evaluate the methods' effectiveness in heterogeneous tissues.

We agree with the reviewer that it will be interesting to evaluate how deconvolution methods perform with rare cell types. In this study, we do not have orthogonal measurements of cell proportions for rare cell types. As mentioned in the discussion:

Future studies could investigate rarer cell types and finer cell type resolutions using spatial transcriptomics technologies that support higher multiplexing, such as MERFISH and Xenium.

That is, we are excited to see how the development of spatial biology transcriptomic assays will enable single cell spatially-resolved expression measurements, which could be used in the future for benchmarking deconvolution methods. We edited the formatting of the discussion so these ideas will be more noticeable.

**3.** Inconsistencies in figure legends, such as those seen in Fig. S26 and Fig. S4, should be corrected. For example, the titles for the y-axis are missing in Fig.S4. Ensuring clarity and consistency in figure legends is necessary to facilitate clear understanding of the data.

We thank the reviewer for their attention to detail, we have added the missing y-axis label “Principal Component Value” to Fig. S4 and improved the figure caption. Fig. S26 was updated and improved with the addition of the HVG analysis discussed earlier.

[**Fig S4**](https://docs.google.com/document/d/1Rk5Z8iyekZoVaYxqjl4nIHx9wTo4p8GjR1J1vki-9Ow/edit#sfigu_bulk_PCA_qc)**: Bulk RNA-seq data Principal Component Analysis (PCA). A.** PC1 versus 2 colored by sequencing round, point shape shows the library type and RNA extraction method **B.** Boxplots of the first six principal components (y-axis) by library type (x-axis) and sequencing round (color).

# Reviewer 3

The authors have addressed all my comments, and I believe this benchmark study provides valuable new insights.

However, I have one remaining concern regarding **Supplemental Figure 26B**, which was added in response to a comment from the first reviewer. The reviewer raised a valid point that the number of marker genes could impact the methods’ performance. One might expect a non-linear relationship, as beyond a certain point, adding more genes may introduce noise without much additional information. **Using a linear regression line based on only four data points as in the figure does not seem adequate to address the reviewer’s question. More data should be presented.**

We are glad our previous changes have satisfied the reviewer and thank them again for the time and attention they have given our manuscript!

We agree with both them and the first reviewer that Fig S26 could be improved with the addition of more datapoints. We have applied an analysis of 10-100% of Highly variable genes that added ten additional evaluations of method performance compared to number of genes. This new analysis is discussed at length above, and has led to an improved Fig S26 as well discussed in response to reviewer 1.

**Third round of review**

**Reviewer 1**

All my previous comments are reasonably addressed.

**Reviewer 3**

The authors have thoroughly addressed all my concerns during the revision process. I believe this is a valuable benchmark study that will assist readers in selecting the most appropriate methods while also highlighting the significant limitations that persist in bulk RNA-seq deconvolution.
